# Methylation of ribosomal RNA by NSUN5 is a conserved mechanism modulating organismal lifespan

**DOI:** 10.1038/ncomms7158

**Published:** 2015-01-30

**Authors:** Markus Schosserer, Nadege Minois, Tina B. Angerer, Manuela Amring, Hanna Dellago, Eva Harreither, Alfonso Calle-Perez, Andreas Pircher, Matthias Peter Gerstl, Sigrid Pfeifenberger, Clemens Brandl, Markus Sonntagbauer, Albert Kriegner, Angela Linder, Andreas Weinhäusel, Thomas Mohr, Matthias Steiger, Diethard Mattanovich, Mark Rinnerthaler, Thomas Karl, Sunny Sharma, Karl-Dieter Entian, Martin Kos, Michael Breitenbach, Iain B.H. Wilson, Norbert Polacek, Regina Grillari-Voglauer, Lore Breitenbach-Koller, Johannes Grillari

**Affiliations:** 1 Department of Biotechnology, BOKU—University of Natural Resources and Life Sciences, Vienna, Muthgasse 18, 1190 Vienna, Austria; 2 Biomedical Sciences Research Complex, University of St Andrews, North Haugh, St Andrews, Fife KY16 9ST, UK; 3 Department of Cell Biology, Division of Genetics, University of Salzburg, Hellbrunnerstrasse 34, 5020 Salzburg, Austria; 4 Christian Doppler Laboratory on Biotechnology of Skin Aging, Muthgasse 18, 1190 Vienna, Austria; 5 Department of Chemistry and Biochemistry, University of Bern, Freiestrasse 3, 3012 Bern, Switzerland; 6 ACIB GmbH–Austrian Centre of Industrial Biotechnology, Muthgasse 11, 1190 Vienna, Austria; 7 Health & Environment Department, Molecular Medicine, AIT Austrian Institute of Technology GmbH, Muthgasse 11, 1190 Vienna, Austria; 8 Department of Chemistry, BOKU—University of Natural Resources and Life Sciences, Vienna, Muthgasse 18, 1190 Vienna, Austria; 9 Science Consult DI Thomas Mohr KG, Enzianweg 10a, 2353 Guntramsdorf, Austria; 10 Department of Molecular Genetics & Cellular Microbiology, Institute of Molecular Biosciences, Goethe University, Max-von-Laue-Strasse 9, 60438 Frankfurt/M, Germany; 11 Biochemie-Zentrum der Universität Heidelberg (BZH), Im Neuenheimer Feld 328, 69120 Heidelberg, Germany; 12 Evercyte GmbH, Muthgasse 18, 1190 Vienna, Austria

## Abstract

Several pathways modulating longevity and stress resistance converge on translation by targeting ribosomal proteins or initiation factors, but whether this involves modifications of ribosomal RNA is unclear. Here, we show that reduced levels of the conserved RNA methyltransferase NSUN5 increase the lifespan and stress resistance in yeast, worms and flies. Rcm1, the yeast homologue of NSUN5, methylates C2278 within a conserved region of 25S rRNA. Loss of Rcm1 alters the structural conformation of the ribosome in close proximity to C2278, as well as translational fidelity, and favours recruitment of a distinct subset of oxidative stress-responsive mRNAs into polysomes. Thus, rather than merely being a static molecular machine executing translation, the ribosome exhibits functional diversity by modification of just a single rRNA nucleotide, resulting in an alteration of organismal physiological behaviour, and linking rRNA-mediated translational regulation to modulation of lifespan, and differential stress response.

## Abstract (web summary)

Cellular pathways modulating longevity and stress resistance are known to affect protein translation. Here the authors show that the RNA methyltransferase, Nsun5, or its yeast homologue Rcm1, regulates lifespan of three different model organisms by modifying ribosomal RNA at a specific cytosine residue.

## Graphical abstract

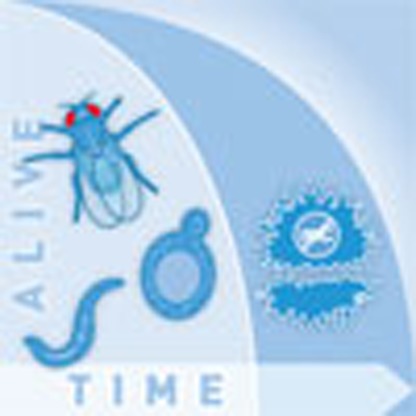

Aging is a complex process characterized by a decline in cellular homeostasis and accumulation of damage that can lead to age-related pathologies. Novel factors and pathways are constantly emerging, but only few are evolutionarily conserved and well understood.

One of these factors is the reduction of overall protein synthesis by either genetic or dietary interventions, which was shown to extend the lifespan in a wide range of different aging models^1^,^2^,^3^,^4^,^5^,^6^,^7^,^8^,^9^. A reduction in protein synthesis and a concomitant increased lifespan were already demonstrated by altering the availability or function of translation initiation factors^1^,^2^,^3^,^5^ or ribosomal proteins^1^,^4^,^6^,^7^,^8^,^9^. However, as it is the case for other interventions designed to counteract aging, also here an enhanced lifespan is coupled to decreased growth and fecundity, supporting the antagonistic pleiotropy hypothesis of aging^10^.

Importantly, the decrease in bulk translation induced by the loss of translation initiation factor eIF4G or dietary restriction is accompanied by an increase in the translation of specific subsets of mRNAs, which is required for the maximal lifespan extension by these interventions^11^,^12^. In addition, oxidative and heat stress were shown to alter specific mRNA recruitment into translating ribosomes^13^,^14^,^15^. Taken together, these and other reports indicate that ribosomes are able to post-transcriptionally modulate the gene expression in response to various environmental stimuli by altering initiation and elongation rates of specific mRNAs with rare codons or *cis*-regulatory elements, such as upstream open reading frames and internal ribosome entry sites^13^,^16^,^17^. Differential expression of ribosomal building blocks, as well as post-transcriptional modifications of ribosomal proteins and rRNAs, then generate ‘specialized’ ribosomes in response to environmental conditions^18^,^19^,^20^. Interestingly, to the best of our knowledge and in contrast to ribosomal proteins and initiation factors, the role of rRNA modifications in aging and ribosomal stress response was not studied so far.

In this report, we identified the RNA methyltransferase NSUN5 to modulate the lifespan and stress resistance of flies, worms and yeast. Using bisulfite sequencing, we could show that the worm and yeast homologues of NSUN5, NSUN-5 and Rcm1, methylate 28S/25S rRNA and that in yeast loss of this methylation induces structural changes that provide altered translating ribosomes under oxidative stress. Importantly, under unstressed conditions we observed in Rcm1-deficient yeast cells a decrease in translational fidelity and an increase in recruitment of stress-specific mRNAs to translating ribosomes, which might explain the increased stress resistance and lifespan.

## Results

### NSUN5 is differentially regulated in cellular aging models

To identify conserved genetic regulators of aging, we recently performed genome-wide comparative transcriptional profiling with selected human-, mouse- and yeast-based aging models. Gene and miRNA expression profiles were measured, analysed, and entered into GiSAO.db (Genes involved in Senescence, Apoptosis and Oxidative stress database)^21^. From this, NSUN5 was shown to be less abundant in senescent kidney cells compared to early passage cells, and in mesenchymal stem cells derived from old and middle aged donors as compared with young donors (Supplementary Fig. 1a,b). Likewise, *RCM1*, the *Saccharomyces cerevisiae* homologue of NSUN5, was downregulated in chronologically aged yeast cells (Supplementary Fig. 1c). Among the differentially regulated genes, NSUN5 caught our special interest because it also physically associates with the PRP19/SNEV-containing complex (personal communication A.L. and J.G.), which we have shown to be involved in the modulation of cellular lifespan and stress resistance in a partially ATM-dependent manner^22^. This prompted us to characterize the function of NSUN5, one of around 30 heterozygously deleted genes on chromosome 7 in Williams Beuren syndrome^23^, in the context of organismal aging.

### NSUN5 modulates the lifespans of model organisms

**Figure 1:**
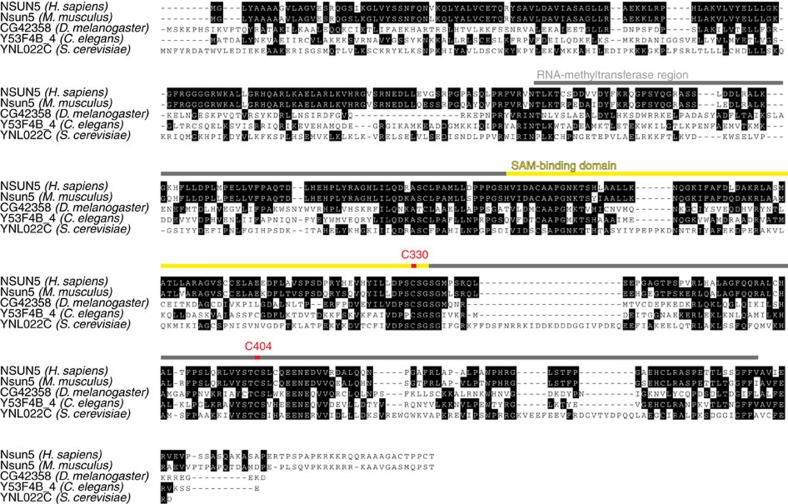
NSUN5 is conserved from yeast to humans. Multiple protein sequence alignment of NSUN5 orthologues from *S. cerevisiae*, *C. elegans*, *D. melanogaster*, mice and humans. Identities are marked with black boxes and functional domains are highlighted.

To study if NSUN5 is causally involved in the aging process, we tested if its downregulation in yeast, worm and fly would modulate their respective lifespans. Therefore, we identified the previously uncharacterized open reading frames CG42358 (NCBI Unigene accession code NP_650787.1) in *Drosophila melanogaster*, Y53F4B.4 (NP_497089.2) in *Caenorhabditis elegans* and YNL022C (NP_014376.3) in *S. cerevisiae* as the closest sequence homologues to the human protein. An alignment of protein sequences of NSUN5 homologues showed high conservation, especially within the functional domain of an RNA methyltransferase (Fig. 1). We propose using the names NSUN5 (Nop2/Sun-like domain containing protein 5) for the human gene, *RCM1* (rRNA cytosine methylase) for the yeast, *dNsun5* for the fly, and *nsun-5* for the worm homologue.

**Figure 2:**
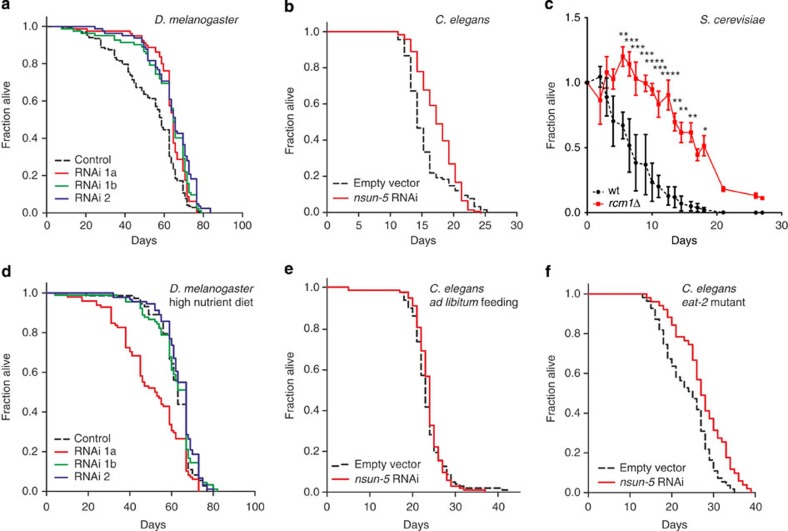
NSUN5 modulates animal lifespan depending on feeding conditions. (**a**) Two individual RNAi constructs of *dNsun5* extend the lifespan of *D. melanogaster* in three RNAi lines (average *N* per group=78, log-rank with Holm–Sidak pairwise comparisons, *P*<0.001 for all three constructs). (**b**) *nsun-5* RNAi in *C. elegans* NL2099 results in increased lifespan (*N*=335, log-rank, *P*<0.001). (**c**) Deletion of *RCM1* in haploid *S. cerevisiae* increases chronological lifespan in water. Error bars represent s.e.m. of three biological replicates (multiple comparison adjusted two-way ANOVA with Sidak post test, *α*=0.05, **P<0.01, ***P<0.001, ****P<0.0001). (**d**) *D. melanogaster* lifespan is not extended by two different *dNsun5* RNAi constructs in three different lines using high yeast and sugar diet (average *N* per group=84, log-rank not significant except RNAi 1a versus control *P*=0.01). (**e**) *nsun-5* RNAi in NL2099 *C. elegans* does not result in increased lifespan when *ad libitum* (AL) fed (average *N* per group=120, log-rank not significant). (**f**) The lifespan of DA1116 harbouring an *eat-2* mutation is further extended by *nsun-5* RNAi (average *N* per group=120, log-rank *P*=0.001).

We first drove ubiquitous expression in male flies with an act5C-Gal4 driver of two different RNA interference (RNAi) constructs in three different RNAi lines targeting *dNsun5*. Remarkably, we observed a robust 16–20% increase in the mean lifespan of all three RNAi lines compared with the control (Fig. 2a and Supplementary Table 1), while no statistically significant differences in size, locomotion or fertility were detected (Supplementary Fig. 2). In contrast, overexpression of *dNsun5* with the same act5C-Gal4 driver significantly reduced mean lifespan by 58% (Supplementary Fig. 3 and Supplementary Table 1).

We next subjected *C. elegans* to *nsun-5* RNAi, while worms were fed *ad libitum* during development, then given single doses of food at the beginning of adulthood and 1 week later. Again, the mean lifespan was significantly extended by 17% (Fig. 2b and Supplementary Table 2). Similar to fly, neither animals exposed to *nsun-5* RNAi, nor the *nsun-5* mutant strain JGG1, showed any significant difference in size, locomotion or pharyngeal pumping (Supplementary Fig. 4).

Finally, we also tested *rcm1* knockout in yeast. We detected an increase in chronological lifespan of *rcm1*-knockout cells compared with wild type (wt), both by analysing survival in water (Fig. 2c), as well as in SC spent medium (Supplementary Fig. 5a). In contrast, we observed a reduction in replicative lifespan (Supplementary Fig. 5b), which is in accordance with reports showing that chronological and replicative lifespans rarely correlate^24^.

### Lifespan modulation by NSUN5 depends on nutrition

The observed increase in lifespan seems to be conditional to media composition and feeding protocols. *dNsun5* RNAi did not increase lifespan in flies fed with a diet that was richer in sugar and yeast (Fig. 2d and Supplementary Table 1). Similarly, *ad libitum* feeding of the worms during the whole life completely abrogated *nsun-5* RNAi-mediated lifespan extension in NL2099 (Fig. 2e and Supplementary Table 2). In accordance with our initial finding, the lifespan of *eat-2* mutant worms, which are dietary restricted due to a decreased pharyngeal pumping rate, was again extended by *nsun-5* RNAi (Fig. 2f and Supplementary Table 2). Similarly, the lifespan of *nsun-5* mutant worms was extended by complete removal of food during adulthood, but not by *ad libitum* feeding (Supplementary Fig. 6). A similar phenomenon may happen in yeast: for chronological lifespan—increased by *rcm1* knockout—cells stay in a very low metabolic state, whereas for division during replicative lifespan cells require and utilize larger amounts of energy. Taken together, these findings suggest that reduced NSUN5 availability only influences lifespan positively when either lower amounts of food, or food with lower energy content is offered. Further studies will clarify this interaction.

### Reduced NSUN5 levels increase resistance to various stresses

**Figure 3:**
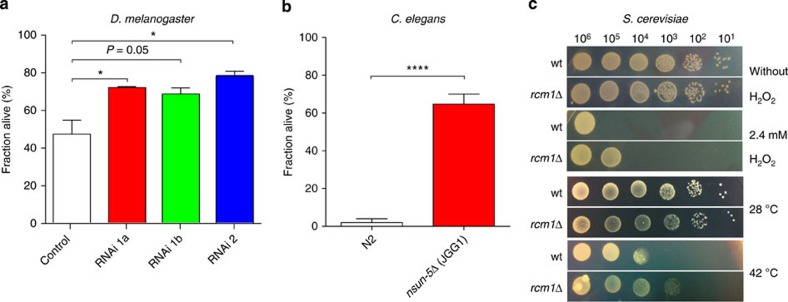
Reduced availability of NSUN5 increases stress resistance of model organisms. (**a**) Flies harbouring two individual RNAi constructs of *dNsun5* in three lines show increased survival compared with control, when subjected to heat shock (4 h at 37 °C, *N*=100, two biological replicates, one-way ANOVA with Dunnett’s post test, *α*=0.05, *P<0.05). Error bars represent s.e.m. (**b**) *nsun-5* mutant worms (JGG1) subjected to 20 mM paraquat treatment for 3 days show significantly increased resistance to the drug (four plates with 30 worms each per genotype, two-sided Student’s *t*-test, ****P<0.0001). Error bars represent s.e.m. (**c**) Yeast serial dilution growth assays demonstrate increased resistance of *rcm1Δ* cells to H_2_O_2_ (upper panel) and to heat shock (lower panel). Uncropped pictures of the entire plates are given in Supplementary Fig. 5.

Increased lifespan is usually associated with increased stress resistance^25^,^26^. Indeed, also in the case of reduced NSUN5, resistance to stress was increased in flies, worms and yeast. Flies were subjected to heat shock and *dNsun5* RNAi strains showed a significantly higher survival on elevated temperature (Fig. 3a). On treatment with paraquat, a generator of reactive oxygen species (ROS), 60% of *nsun-5* mutant worms survived compared with only 2% of the wt (Fig. 3b). Similarly, *nsun-5* RNAi significantly increased the survival on paraquat containing plates of N2 wt worms (Supplementary Fig. 5c). Accordingly, plate assays with *S. cerevisiae* demonstrated an increased resistance of the *rcm1*-knockout strain against H_2_O_2_ (Fig. 3c and Supplementary Fig. 5d), as well as heat shock (Fig. 3c and Supplementary Fig. 5e). Also in liquid medium in the presence or absence of H_2_O_2_, *rcm1*-knockout yeast proliferated under mild oxidative stress as well as unstressed wt cells (Supplementary Fig. 5f). Taken together, these data suggest that the loss of NSUN5 confers increased resistance to different types of stress in a conserved manner.

### NSUN5 is a conserved rRNA methyltransferase

**Figure 4:**
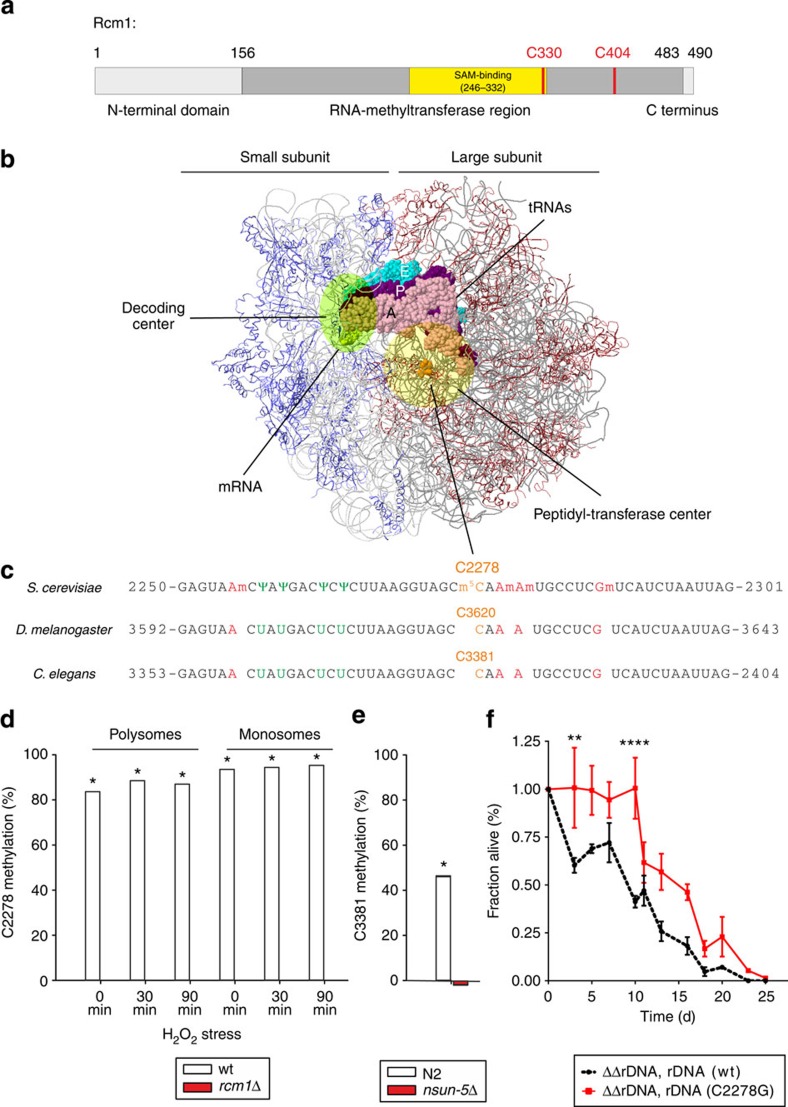
The conserved m^5^C-rRNA methyltransferase activity of Rcm1 mediates lifespan extension. (**a**) Rcm1 is a protein of 490 amino acids harbouring an RNA methyltransferase domain with two highly conserved cysteines, C330 and C404, which are predicted to participate in the catalysis of methyl transfer. (**b**) Model of the yeast ribosome with functional elements. The large and small ribosomal subunits, RPs of the SSU in blue and of the LSU in red, are shown assembled on the mRNA, the tRNAs in the aminoacyl site (A, pink), in the peptidyl site (P, magenta) and in the exit site (E, cyan) are depicted, and cytosine 2278 (C2278) of the 25S rRNA in vicinity of the peptidyltransferase centre is highlighted in orange. (**c**) 25S rRNA sequence tract, nucleotide 2251 to 2300, harbours a series of highly conserved modified nucleotides, pseudouridinylations in green and guanine and adenine base methylations in red, and the single m^5^C-methylation in orange. This region is 100% conserved from *S. cerevisiae* to *C. elegans* and *D. melanogaster*. (**d**,**e**) Bisulfite sequencing of wt yeast cells detects C2278 methylation in rRNA isolated from ribosomal fractions independent of oxidative stress. Deletion of *rcm1* resulted in a complete lack of cysteine C2278 methylation under all conditions tested (**d**). Bisulfite sequencing of N2 wt *C. elegans* confirms conservation of C3381 m^5^C-methylation, while deletion of *nsun-5* resulted in a complete lack of this modification (**e**). Displayed values represent fraction of C2278/C3381 methylation minus average fraction of unconverted Cytosines except C2278/C3381 as unspecific background. Grubbs’ Test at *α*=0.01 was performed on all Cytosines in the sample and asterisk * marks samples with C2278/C3381 identified as significant outlier. (**f**) Deletion of ribosomal DNA in haploid *S. cerevisiae* and rescue with unmethylatable rDNA (C2278G) on a plasmid increases chronological lifespan in SC compared with wt rDNA (wt). Error bars represent s.e.m. of three biological replicates (multiple comparison adjusted two-way ANOVA with Sidak post test, *α*=0.05, **P<0.01, ****P<0.0001).

We next investigated the molecular mechanism by which NSUN5 modulates lifespan and stress resistance, using yeast. Rcm1 has an m^5^C-RNA methyltransferase domain with two highly conserved cysteines (C330 and C404) forming the active centre^27^,^28^, as well as an *S*-adenosyl-methionine (SAM)-binding pocket (Fig. 4a). Rcm1 localizes to the nucleoli^28^,^29^ and interacts with the methyltransferase adaptor protein Trm112 (ref. 30). Human NSUN5 also co-precipitates with ribosomes^31^. These findings all suggest a role in rRNA modification during early ribosome biogenesis. Indeed, Sharma *et al.* and Gigova *et al.*^28^,^32^ recently demonstrated that Rcm1 directly methylates cytosine 2278 (C2278) of 25S rRNA. C2278 is located in an rRNA region neighbouring several other highly conserved modified rRNA nucleotides with 100% sequence conservation from yeast to *C. elegans* and *D. melanogaster*. This region is located in close proximity to the peptidyltransferase centre of the large ribosomal subunit (Fig. 4b,c)^33^, suggesting that it is important for core ribosomal function. We decided to confirm that Rcm1 is indeed responsible for methylating C2278 and to test if this methylation might be ROS dependent.

To this aim, we performed comparative bisulfite sequence analysis from wt and *rcm1*-knockout strains and also included RNA from cells stressed with H_2_O_2_ (0.4 mM) for 30 min and 90 min, respectively. In *rcm1*-knockout cells, C2278 was not methylated under all conditions tested, while in wt cells ~80% of rRNA isolated from ribosomal gradients was methylated at C2278, independent of oxidative stress (Fig. 4d and Supplementary Fig. 7). Thus, we conclude that Rcm1 is necessary and sufficient for the formation of a large C2278-methylated subpopulation of ribosomes, both under normal and oxidative stress conditions.

By applying the same method to *C. elegans*, we could prove that C3381, which corresponds to yeast C2278, is indeed methylated by *nsun-5* (Fig. 4e). Thus, not only the modifying enzyme, but also the target site and probably molecular mechanism are highly conserved between these two organisms.

To demonstrate that methylation of C2278 and no other molecular activity of Rcm1 is required for Rcm1-mediated lifespan extension, we performed chronological lifespan assays with yeast strains in which all rDNA repeats have been deleted. This loss of rDNA was complemented with plasmids carrying either wt rDNA, or rDNA with a point mutation exchanging C2278 to non-methylatable G2278 (ref. 28). Indeed, the strain carrying the C2278G rDNA plasmid showed a significantly extended chronological lifespan (Fig. 4f). These data suggest that the lack of NSUN5 methylation activity towards a single nucleotide of rRNA is indeed sufficient for the observed lifespan modulation.

### Loss of Rcm1 leads to structural changes of rRNA on stress

**Figure 5:**
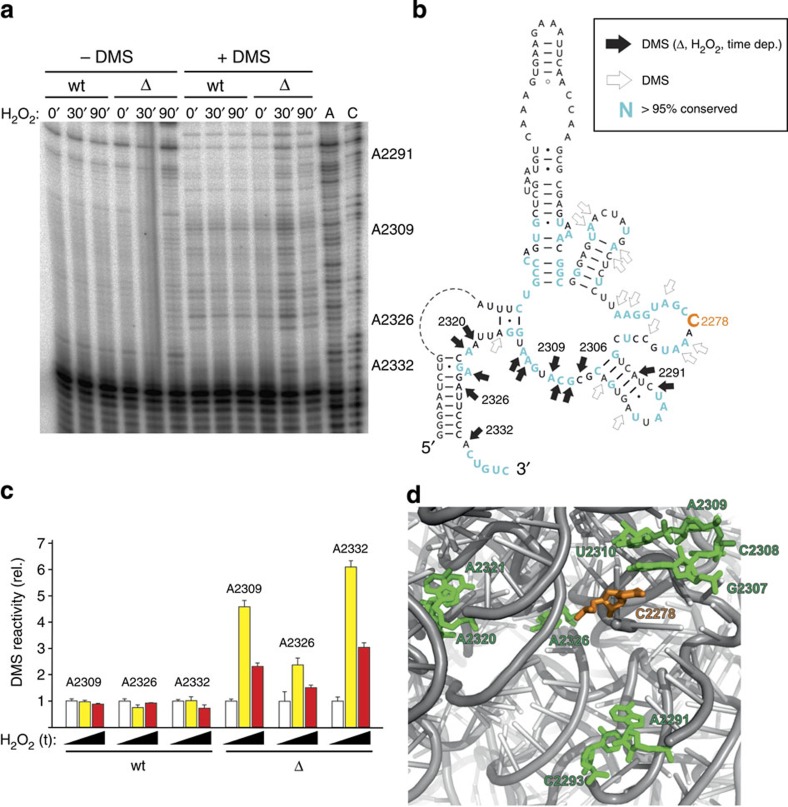
Loss of Rcm1 leads to structural changes of rRNA on oxidative stress. (**a**) Primer extension analysis of 25S rRNA of DMS-treated ribosomes. Ribosomes from wt and *rcm1*-knockout cells (Δ) were treated with H_2_O_2_ for 0, 30 or 90 min and subsequently analysed for DMS-reactive adenosines and cytidines. Note that the reverse transcriptase stops one nucleotide 3′ of the modified nucleobase. A and C denote dideoxy-sequencing lanes of untreated 25S rRNA and serve as marker for identifying DMS-reactive sites at the nucleotide resolution level. The positions of some DMS-reactive adenosines are indicated on the right. (**b**) Secondary structure diagram of the 25S rRNA region surrounding position C2278 (orange). DMS-reactive sites that change their reactivity only in the knockout strain during stress in a time-dependent manner are indicated by solid arrows. Additional positions that can be modified by DMS but do not show a differential pattern are indicated by open arrows. Nucleobases that are universally conserved (>95% conservation) in all three domains of life as well as in chloroplasts and mitochondria are highlighted in cyan. (**c**) Quantification of the DMS reactivity at selected rRNA positions in wt and *rcm1*-knockout (Δ) cells. The primer extension stop intensities of DMS-reactive nucleobases in the absence of H_2_O_2_ was taken as 1.0 and compared with the corresponding bands in ribosomes treated with H_2_O_2_ for 0 (white bars), 30 min (yellow bars) or 90 min (red bars). Values shown represent the mean and the s.d. of three independent DMS-probing experiments. (**d**) Three-dimensional representation of the structural proximity of C2278 (shown in orange) with DMS-reactive residues that showed a temporal enhanced accessibility after 30 min of H_2_O_2_ stress (green). The three-dimensional architecture was generated from pdb file 35UI.

A recent report suggests that the loss of a cluster of methylations including C2278 leads to structural changes and ribosome instability under high-salt conditions^32^. To investigate if there are any conformational changes of the ribosome as a consequence of the loss of Rcm1 function alone, we performed structural probing employing the single-strand specific chemical probe dimethyl sulfate (DMS). Ribosomes isolated from yeast wt or from *rcm1*-knockout cells were analysed in the presence or absence of oxidative stress (Fig. 5). While after 30 min of oxidative stress, some universally conserved nucleotides surrounding position C2278 became more accessible to DMS in the absence of Rcm1, this effect diminished after 90 min (Fig. 5). Thus, the loss of Rcm1-mediated methylation of C2278 leads to a transient structural change in the presence of H_2_O_2_, likely leading to a more relaxed 25S rRNA fold in the vicinity of C2778.

### Methylation by Rcm1 modulates the function of ribosomes

**Figure 6:**
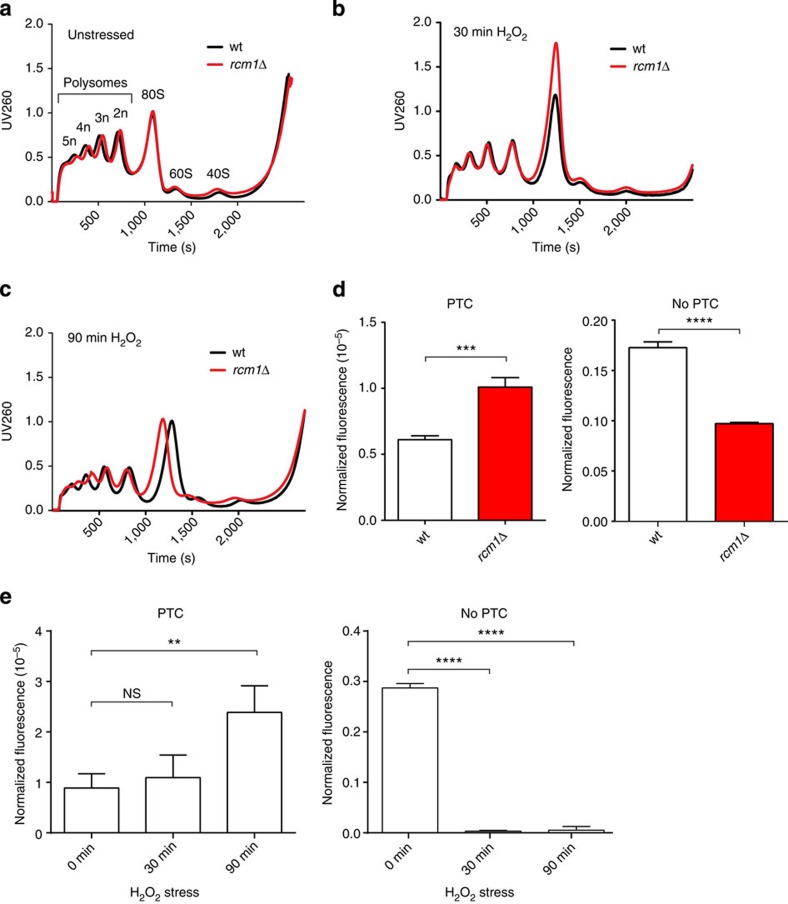
Rcm1 mediates changes in ribosome profiles and stop codon read-through. Ribosomal profiles provide a snapshot of the translational status and are obtained by UV260-tracing of polysomes, single 80S ribosomes and free LSU and SSU populations, respectively, separated on a continuous sucrose gradient. (**a**) Loss of Rcm1 does not alter the ribosomal profile representing bulk translation. (**b**) Both in wt and Rcm1-deficient cells, 30 min H_2_O_2_ stress induces a shift in ribosomal profile subpopulations, with decrease of polysomes and increase of 80S. (**c**) After 90 min of H_2_O_2_ exposure, the decrease in polysome population remains, with the 80S peak recovering to a wt-like recording. (**d**,**e**) Loss of Rcm1 (**d**) as well as oxidative stress (**e**) significantly promote reading through a PTC mutation within a FF reporter construct (*N*=6, two-sided Student’s *t*-test **P<0.01, ***P<0.001, ****P<0.0001). FF luminescence, representing PTC read-through, was normalized to REN luminescence, representing transfection efficiency. Reporter luminescence without a PTC and normalized as above is also shown (no PTC). In this case, Rcm1 deletion and oxidative stress decrease normalized reporter luminescence.

We next investigated which functional changes occur in translating ribosomes as a consequence of the Rcm1-mediated structural alterations of rRNA. Therefore, we performed polysome profile analysis, which provides a snapshot of the translationally active ribosome population^34^. Interestingly, comparison of the profiles obtained from wt and *rcm1*-knockout cells did not show differences in the distribution of polysomes, 80S monosomes and free subunits, as previously reported^28^ (Fig. 6a and Supplementary Fig. 8).

However, as *rcm1*-dependent conformational changes of the ribosome occur only on oxidative stress, we tested if also the polysome profiles were influenced by ROS using a single-dose treatment of 0.4 mM H_2_O_2._ After 30 min, the stoichiometry between the actively translating ribosome population, that is, the polysomes, was shifted towards the 80S monosome peak as reported^15^, both in wt and rcm1-knockout cells. Of note, the increase in the 80S monosome peak in *rcm1*-knockout cells was by 33% more pronounced (Fig. 6b and Supplementary Fig. 8). This suggests that the presence of C2278 methylation in wt cells limits, while loss of C2278 methylation in *rcm1*-knockout cells intensifies the shift from polysomes towards more 80S monosomes on acute stress. After 90 min of H_2_O_2_ treatment, the ribosomal profiles of *rcm1*-knockout cells resembled those of the wt, both approaching the unstressed status again (Fig. 6c). These results are in line with the DMS-probing results above, showing the main difference in the ribosome structure only 30 min after exposure to ROS, and suggest that the Rcm1-dependent methylation of C2278 is important for a balanced ribosomal response to acute stress.

To investigate the functional consequences that may result from lack of C2278 methylation, we tested translational fidelity of ribosomes lacking C2278 methylations using a luciferase reporter construct with a premature termination codon (PTC). Rcm1 knockout promoted read-through of the premature stop codon by a factor of 2, compared with wt cells. Expression of the same reporter lacking a PTC (no PTC) was reduced in Rcm1-knockout cells (Fig. 6d). This resembled increased read-through of the PTC reporter induced by oxidative stress in wt yeast (Fig. 6e).

### Rcm1 reprograms the translational oxidative stress response

Since it has been previously reported that oxidative stress alters the fidelity of mRNA translation, increasing misreading and stop codon read-through^13^,^16^, these results suggest that the absence of C2278 methylation resembles an activated cell status that counteracts oxidative stress. To test this idea, we analysed the mRNA recruitment pattern into polysomes versus total cellular mRNAs under different oxidative stress conditions by microarrays.

**Figure 7:**
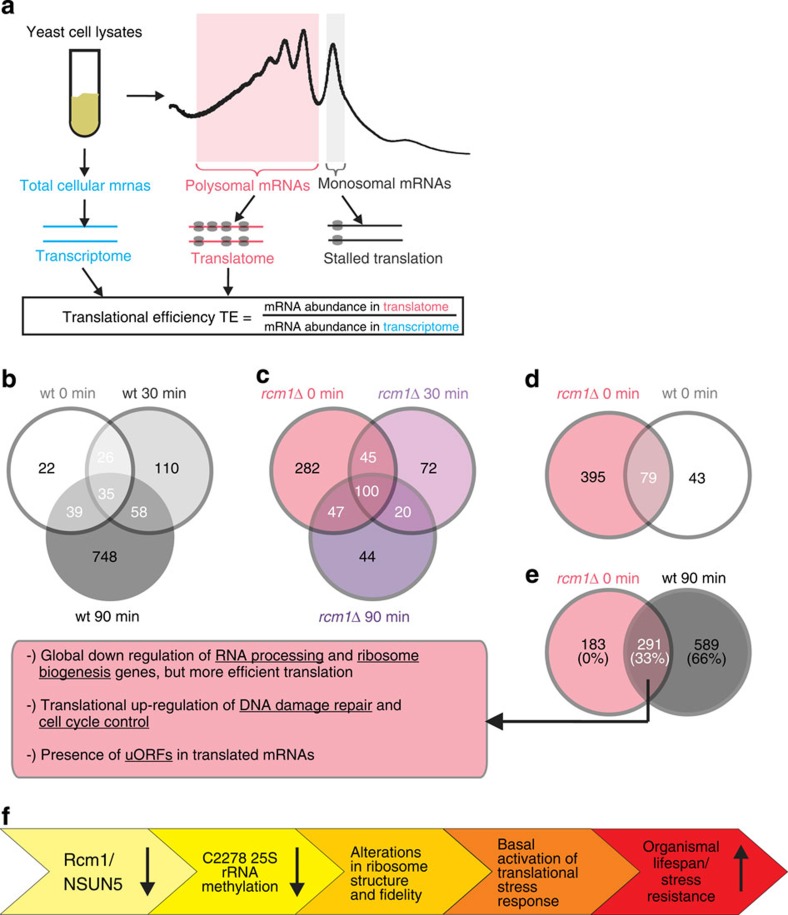
Stress-responsive mRNA translation is constitutively activated on loss of Rcm1. (**a**) Diagrammatic representation of genome-wide translational profiling. Expressed total mRNAs (transcriptome) and actively translated mRNAs within polysomes (translatome) were isolated from whole yeast cell lysates and analysed on microarrays in biological duplicates. TE is the ratio of the expression of a specific gene in the translatome versus the transcriptome. (**b**–**e**) Venn diagrams comparing the number of genes, which are differentially translated under different oxidative stress conditions and genotypes (*P*<0.05, log2(TE)>1.5 or <−1.5). wt and *rcm1* knockout (*rcm1Δ*), as well as unstressed (0 min), 30 and 90 min H_2_O_2_ (0.4 mM) are depicted. Most of the genes differentially translated on *rcm1* knockout are also regulated on 90 min of oxidative stress in wt. (**f**) We hypothesize that the availability of Rcm1, and thus C2278 methylation levels, modulate lifespan. In this scenario, the reduced availability of Rcm1 causes reduction of C2278 methylation and structural alterations in the adjacent rRNA region. This alters the translational fidelity accompanied by mRNA recruitment in a similar fashion to oxidative stress, leading to increased stress resistance and lifespan.

To estimate the efficiency of translation of individual mRNAs, we calculated the translational efficiency (TE) as ratio between signal intensity in the polysome fraction (‘translatome’) versus signal intensity in the total yeast cell lysate (‘transcriptome’) for each probe set on the array (Fig. 7a). Genes with at least twofold up- or downregulation at *P*<0.05 (multiple comparison adjusted *t*-test) were defined to be significantly translationally up- or downregulated.

We found 122 genes to be translationally regulated in untreated wt cells, while after 90 min of oxidative stress the efficiency of translation of 880 genes was significantly different, with only a small overlap of genes in all untreated, 30 and 90 min stressed cells (Fig. 7b, Supplementary Data 1–2). In *rcm1*-knockout cells, however, already under unstressed, basal conditions a high extent of translational regulation of gene expression (474 genes) was observed, suggesting that the lack of C2278 methylation specializes the ribosome complement for recruitment of different mRNAs out of the offered pool of totally transcribed mRNAs. Then, under stress, a blunted response was observed and fewer mRNAs (211 genes for 90 min of H_2_O_2_) were differentially recruited to the polysome fraction (Fig. 7c, Supplementary Data 1–2). Remarkably, of those 474 genes in unstressed *rcm1*-knockout cells, only 79 genes were similarly regulated in unstressed wt cells (Fig. 7d, Supplementary Data 1–2). However, 291 out of these 474 genes were also under translational control in 90 min stressed wt cells (Fig. 7e, Supplementary Data 1–2). Thus, one-third of all genes that are translationally regulated by H_2_O_2_ in wt cells, are already differentially expressed by *rcm1* deletion. We noticed that many of these mRNAs have short upstream open reading frames within their 5′ UTRs, including several already previously published^16^, which are known to modulate gene expression on translational level in both directions^13^,^16^.

Gene set enrichment revealed a significant (false discovery rate<25%) translational upregulation of pathways linked to cell cycle control and DNA damage response, both in stressed wt cells, as well as in unstressed *rcm1* knockouts (Supplementary Data 3). Counterintuitively, under these conditions also, gene sets associated with ribosome biogenesis were strongly and significantly overexpressed. To further investigate this surprising finding, we compared stressed wt cells and *rcm1* knockouts with unstressed wt cells on transcriptome and translatome level individually. We found that ribosome biogenesis was strongly downregulated both on transcriptome and on translatome level on stress and loss of Rcm1. Therefore, we conclude that both on *rcm1* knockout as well as under oxidative stress, reduced levels of mRNAs of genes involved in ribosome biogenesis are present, but more efficiently translated. This mechanism would allow the cells to faster modulate ribosome biogenesis and thereby also protein synthesis in the event of stress. Interestingly, we observed that after 30 min of stress, the gene set of ‘cytoplasmic translation’ was significantly and strongly translationally downregulated in *rcm1*-knockout cells, but not in wt. This finding is in accordance with the previously observed increase of 80S monosomes compared with polysomes in *rcm1*-knockout cells on 30 min of stress.

Taken together, our translational profiling data suggest that the loss of *rcm1* leads to a more efficient translation of stress-responsive mRNAs, even under unstressed conditions. Thus, preactivation of the translational stress response by *rcm1* deficiency and the resulting improved modulation of cytoplasmic translation on stress, might contribute to the increased stress resistance and longevity of the NSUN5-deficient model organisms studied (Fig. 6f).

Interestingly, we also found that both on transcriptional and translational level, gene sets associated with RNA methylation and maturation were always significantly downregulated in *rcm1*-knockout cells in comparison with their respective wt controls. This suggests a feedback loop, in which the loss of a single-RNA-modifying enzyme (Rcm1) induces a global downregulation of RNA processing.

We confirmed the microarray results by comparing the expression values from a subset of genes from the array, including the top regulated genes previously published by Gerashchenko *et al.*^13^, with three independent biological replicates by quantitative PCR (qPCR) and observed similar trends in gene expression (Supplementary Fig. 9).

## Discussion

Here we describe that the loss of NSUN5 is a conserved mechanism increasing the lifespan and stress resistance of yeast, flies and worms, which is mediated by the lack of methylation of a single rRNA nucleotide. This is so far one of the rare examples of direct physiological effects of a ribosomal RNA modification. Although a point mutation of C2278 of 25S rRNA, which makes methylation impossible, is sufficient to confer an increased chronological lifespan in yeast (Fig. 4f), NSUN5 might have additional methylation substrates which might also contribute to the observed phenotypes. Thus, the identification of additional NSUN5 methyltransferase target sites by recently developed techniques, such as Aza-IP^35^, might be of great interest.

Remarkably, reduced levels of NSUN5 as assessed by polysome profile analysis did not decrease bulk protein translation (Fig. 6a and personal communication M.S. and J.G.), which is already known to extend lifespan of various model organisms^1^,^2^,^3^, probably by allocating energy from reproduction to maintenance and damage repair^10^. However, a specific protein, namely, the overexpressed luciferase reporter protein without a PTC was less efficiently translated on loss of Rcm1, as well as under oxidative stress (Fig. 6e). Although luciferase is no endogenous yeast gene, this might indicate that specific proteins, such as the PTC-less luciferase reporter, are differentially translated. Even if the reporter is ectopically overexpressed and thereby might overload the translational machinery, this finding would be in line with the observation that loss of NSUN5, as well as oxidative stress, specifically control recruitment of stress-responsive mRNAs into polysomes (compare Fig. 7b–e). These specific mRNAs include factors involved in cell cycle control and DNA damage repair, among others. While an upregulation of cell cycle control proteins arrests the cell to avoid propagation of damage during DNA synthesis and mitosis, the increased amounts of DNA damage repair factors and regulators might help to sense and repair diverse lesions, before resuming the cell cycle.

Our results on the differential recruitment of stress-responsive mRNAs support the concept of specialized ribosomes. First, although oxidative stress induces substantial translational reprogramming of gene expression both in wt and *rcm1*-knockout cells, there is a drastic difference in the pattern of recruited mRNAs between the two genotypes. While wt cells show the highest degree of translational regulation after 90 min of oxidative stress, *rcm1*-knockout cells display *ab initio* extensive translational reprogramming, and then a blunted response to stress. Thus, we propose that the unique oxidative stress response on loss of *rcm1* arises from the presence of a cellular complement of redox-sensitive specialized ribosomes that carry unmethylated C2288. Second, the expression of 23 genes is translationally regulated under every condition tested and we therefore conclude that this subset represents differentially translated proteins necessary for obligatory cellular functions, regardless of the genotype or stress. However, only eight genes are commonly regulated both in wt and *rcm1*-knockout cells after 30 min, and 20 genes after 90 min of stress, respectively. This small overlap documents that only a minor fraction of the overall translational stress response acts independently of Rcm1 function and further emphasizes that the Rcm1-mediated response is distinct from that of wt cells. Further analysis of these data sets to clarify the individual roles of selectively expressed stress-responsive proteins will be presented elsewhere.

Since NSUN5 transcription was found to decrease in cellular model systems during aging under physiological conditions, as a consequence lower levels of C2278 methylation will favour more efficient translation of stress-response proteins. Elevated levels of these might consequently enable cells to better tolerate oxidative stress and prolong lifespan. Alternatively, this might be part of a stress-defence memory of cells that have been repeatedly exposed to stress during their lifespan and will therefore be more resistant during consecutive encounters and thus live longer. However, we cannot exclude the possibility that lack of NSUN5 poses a (beneficial) stress to the cells and thus indirectly increases the recruitment of stress-related mRNAs.

By any of these depicted mechanisms, knockdown of NSUN5 extends lifespan and stress resistance without affecting body size or fecundity of flies and worms, while most of the known genetic and nutritional interventions extending the lifespan of organisms also antagonistically decrease growth, body size and fecundity^10^,^36^. Thus, it remains elusive why evolution selected for the presence of NSUN5, which shortens lifespan on reduced nutrient availability and has so far no observable beneficial effects. It seems likely that the loss of NSUN5 also has detrimental effects, for example, the reduced replicative lifespan of yeast mother cells, the reduced translational fidelity, or other not yet identified ones, which appear only under certain environmental conditions. Probably, these detrimental effects counteract beneficial ones and are neutralized by mild dietary restriction, which leads to the observed lifespan extension only under reduced nutrient availability.

Our observation that NSUN5-mediated lifespan extension is dependent on lower amounts of food, or food with lower energy content, might also be explained by a putative link between NSUN-5 activity and nutrient availability through nutrient sensing pathways, such as TOR, or *sams-1*. SAMS-1 is required for the synthesis of the universal methyl group donor SAM in *C. elegans* and is downregulated on dietary restriction^37^. Furthermore, *sams-1* RNAi extends worm lifespan^37^, which might be partially mediated by lack of substrate for NSUN-5 and other RNA methyltransferases. Apart from this study on NSUN5, Curran and Ruvkun^5^ identified two other RNA methyltransferases extending *C. elegans* lifespan; however, they remain functionally uncharacterized so far.

Therefore, further studies aiming at the identification of upstream regulators of NSUN5 activity, especially in response to dietary conditions, are required to fully understand the biological function of NSUN5 and significance of C2278 methylation, which led to the high evolutionary conservation of both.

Our finding that cell cycle control proteins are translationally upregulated on *rcm1* knockout in exponentially growing yeast cells suggests that loss of Rcm1 induces a translational response, which might vary depending on the growth phase. Furthermore, the reduction of replicative lifespan might be a consequence of the activation of proteins involved in the negative regulation of the cell cycle. Thus, our proposed molecular mechanism underlying NSUN5-mediated stress resistance and longevity, which we conclusively tested in replicating yeast, needs to be validated in non-replicating yeast. However, ribosomal profiles from stationary phase cells closely resemble those of oxidatively stressed wt cells (personal communication L.B.-K.), so it would not be possible to directly compare the translational status of differentially stressed or *rcm1*-knockout cells. Moreover, translational fidelity and mRNA recruitment are also altered by entry into stationary phase (personal communication L.B.-K.). Analysis of this matter in detailed genetic and biochemical assays is needed and will be the scope of further studies, as well as testing our proposed molecular mechanism in *C. elegans* and *D. melanogaster*. This might prove to be technically demanding, since methods for testing ribosome conformation and fidelity are not yet well established in these organisms.

Finally, our data together with previous reports suggest that the cellular reaction to various signals, such as nutrient availability^3^, oxidative stress^13^,^15^,^17^ and heat shock^14^, converge on changing ribosomes not only in terms of ribosomal proteins, their modification or stoichiometry, but also in terms of rRNA modifications. In the case of NSUN5, this change probably occurs already during ribosome biogenesis, since Rcm1 was reported to localize to the nucleoli^28^,^29^, where rRNA is transcribed and processed and preribosomal particles are assembled^19^. Thus, the NSUN5-mediated stress response is probably slow, since it depends on the exchange of methylated with unmethylated ribosomes. This leads us to propose that NSUN5 activity is preferentially modulated by long-term or chronic stress, as during the aging process. Thus, the C2278 unmethylated and thereby ‘specialized’ ribosome subpopulation, which is already present in young and unstressed cells (compare Fig. 4d), will hypothetically grow larger during aging and chronic stress. These specialized ribosomes have the unique ability to sense stress and consequently alter their structural confirmation, as well as fidelity. This enables them to selectively recruit and translate mRNAs to react to distinct cellular signals, such as various stressors, in a fast and efficient way^18^,^19^,^20^.

In summary, we propose that NSUN5 contributes to the generation of stress-counteracting specialized ribosomes, which participate in extending the life- and healthspan of cells and organisms. It is tempting to speculate how stress-counteracting ribosomes might be employed to improve health conditions of an aging society.

## Methods

### General statistics and experimental design

Sample sizes were estimated from literature for the respective assays. No systematic randomization method was used, but samples were never prepared and analysed in a predefined order. No systematic blinding throughout all assays was done, but at least one replicate of all lifespan- and stress resistance assays was performed with similar outcome by a different operator, who was blinded to the group allocation.

### Fly strains and culture conditions

The UAS-CG42358 (*dNsun5*) RNAi flies were obtained from the Vienna Drosophila RNAi Center^38^. The sequences for shRNAs are as follows:

*RNAi 1a (179H1.T1):*

5′-TCCAACTACTGGGTCCGCCATCCGTTGGTGCACAGCAAGAGGTTTATACTGCAGAACAAGGCCACCTGTTTGGCCGCCGAACTACTTGCTCCGCCGTCT GGGGCCACTGTGCTGGATATGTGCGCAGCTCCCGGTATGAAGACAGTGCACATATGCAATGTGATGCAGAACAAGGGATGCATCTACTCCGTGGAGCAGGACCACGTACGCTACAATACGCTGTGCGAAATAACAAAGGATGCTGGTTGC GATATTGTAAAACCCATTTTGGGAGACGCCTTGAACCTAACACCCGAACGCTTTCCGGACGTAGAGTACATCCTGGTGGACCCTAGTTGCTCTGGCAGCGGAATGCAAAACCGCATGACCGTGTGCGACGAGCCGAAGGAGGACAAGC-3′ (inserted into chromosome 3).

*RNAi 1b (179H1.T2):*

Same as RNAi 1a, but inserted into chromosome 2.

*RNAi 2 (32E11.T2):*

5′-GGAGGACAAGCGGCTGCAAAAGCTGCAGGGTTTGCAAATTAAGATCCTGTCGCACGCGATGGGCGCCTTTCCGAACGTCAAACGCATTGCCTACTGCACGTGTTCGCTGTGGAAGGAGGAAAACGAGCAGGTGGTGCAGCGTTG TCTTCAGCTAAATCCATCCTTCAAGCTGCTCAGCTGCAAGAAGGCCTTGCGCAACAAGTGGCACAATGTGGGCGACAAGGACTATCCCAATATTGGCAAGAACGTCCTGTATTGCCAGCCGGACAGTGATCTTACCGATGGCATCTTCCTGGCCCTTTTCGAGA-3′ (inserted into chromosome 2).

The line overexpressing *dNsun5* was obtained from the Drosophila Genetic Resource Center. The lines were crossed to an act5C-GAL4 driver and the progeny of the cross carrying both the RNAi (or overexpressing) and the GAL4 constructs were the experimental flies. The control flies were the progeny of the cross between the lines used to create the transgenic lines (w^1118^ for the RNAi lines and y[1] w[67c23] for the lines overexpressing dNsun5) and the GAL4 driver carrying both the transgenic and GAL4 constructs.

All flies used in experiments were mated once and collected soon after emergence unless specified otherwise. Flies were kept on a 2.2% sugar–8% corn flour–1.8% yeast diet enriched with live yeast at 25 °C on a 12L/12D photoperiod unless specified otherwise.

### Longevity measurement of flies

Figures show representative data of two independent biological replicates. Flies were collected and placed in plastic vials, 20 males in each vial. Around 100 flies were used in each group for each replicate. Dead flies were counted every weekday until the death of the last fly. They were transferred to fresh food twice a week. For measurement on richer food, flies were kept on a 6% sugar–2% peptone–10% yeast diet enriched with live yeast. The Kaplan–Meier survival curves were computed and compared with log-rank tests implement in SigmaPlot version 11 (Systat Software). Lifespan measurements were carried out on both RNAi flies and flies overexpressing *dNsun5*.

### Resistance to heat of flies

Twenty-one-week-old males were transferred into a sealed vial in which a paper filter soaked with water had been inserted for a total of 100 flies per group and replicate. The vials were placed for 4 h in a water bath at 37 °C. At the end of the heat shock, flies were transferred into vials with fresh food and incubated at 25 °C. The number of dead flies was counted 24 h after the end of heat shock. The data of two biological replicates was analysed by one-way analysis of variance (ANOVA) with Dunnett’s post test using GraphPad Prism version 5.00 for Windows (GraphPad Software).

### Size measurement of flies

Ten males and 10 females per group (control and *dNsun5* RNAi) were anaesthetized under light CO_2_ and lined up under a stereomicroscope, upwards facing. Pictures of the thorax were taken, all at the same magnification, and the length of the thorax was measured from the pictures (length expressed in number of pixels). The data of males and females were individually analysed by one-way ANOVA with Dunnett’s post test using GraphPad Prism version 5.00 for Windows (GraphPad Software).

### Locomotor activity of flies

Forty males were collected for each genotype with 10 flies per vial. At 12, 19, 26 and 47 days of age, flies were transferred into empty vials and their climbing activity was measured in two different ways:

First, each vial was placed on a shaker and a gentle shake was given for all the flies to fall to the bottom of the vial. Then the number of flies able to reach the top of the vial (6 cm) in 18 s was recorded. The measurement was repeated three times for each vial. The percentage of flies able to reach the top of the vial was computed and the effect of age and genotype was assessed with a two-way ANOVA with Bonferroni post tests using GraphPad Prism version 5.00 for Windows (GraphPad Software).

In a second measurement done on the same flies, we used a 3-s mechanical stimulation and 4 s after the end of the stimulation, a picture was taken with a digital camera. The height reached by each fly was then recorded. In this measurement, the effect of age and genotype on the distance climbed by each group was tested with a two-way ANOVA with Bonferroni post tests as before.

### Fertility of flies

Twenty virgin females were collected for each genotype and put singly into vials with two males of the same genotype. Flies were transferred into new vials three times a week and the vials were checked for dead flies. The empty vials were kept at 25 °C and 10 days later the number of pupae in each vial was counted for 4 weeks. The vials in which females were still present at the end of the 4 weeks, the mean number of pupae per female of the *dNsun5* RNAi lines was compared with the control line by One-way ANOVA with Dunnett’s post test using GraphPad Prism version 5.00 for Windows (GraphPad Software).

### Worm strains and culture conditions

Strains were cultured under standard laboratory conditions on *E. coli* OP50-seeded NGM agar plates at 20 °C (ref. 39). Strains used in this work include N2 (provided by V. Jantsch), JGG1 (*nsun-5*(tm3898) II), NL2099 (*rrf-3*(pk1426) II) and DA1116 (*eat-2*(ad1116) II). All strains are available through the Caenorhabditis Genetics Center.

### *nsun-5* mutant strain (JGG1) generation

The JGG1 *nsun-5* mutant strain was generated by backcrossing six times FX03898 (*nsun-5*(tm3898) II; generously provided by S. Mitani and the *C. elegans* gene knockout consortium) against N2. The presence of the deletion leading to the tm3898 allele was verified throughout the whole backcrossing procedure by PCR.

### RNAi knockdown of gene expression in worms

For the inactivation of NSUN-5, feeding of double-stranded RNA expressed in bacteria was used^40^. Therefore, the HT115 strain of *E. coli* carrying the RNAi construct or the empty vector (L4440) was cultured overnight in liquid LB with ampicillin and tetracyclin at 37 °C. The bacteria were harvested by centrifugation, resuspended in LB to a concentration of 60 mg ml^−1^ and 200 μl of this suspension was streaked out on NGM plates containing 1 mM isopropyl-β-D-thiogalactoside and 25 μg ml^−1^ Carbenicillin. The plates were incubated at 37 °C overnight and used within one week.

### Lifespan measurement of worms

Lifespan assays were performed following standard protocols^1^. Figures show representative data of at least two independent biological replicates. Worms were cultured on either ultraviolet-killed OP50-seeded NGM plates (for assay N2 versus JGG1) or plates containing the respective RNAi bacteria for at least two generations before the actual experiment. Synchronous cultures were obtained by transferring 15–25 young adult nematodes on plates and letting them lay eggs for 4 h. When the progeny reached adulthood, they were transferred to fresh NGM plates containing 40 μM FuDR. This day was recorded as day 0. Nematodes were scored as dead or censored every day. Censored animals include worms that crawled off the plate or died from causes other than aging such as gonadal extrusion or internal hatching of progeny. The number of censored animals was always comparable to the controls within experiments. Animals were transferred to fresh plates every 3–7 days depending on the availability of bacteria on old plates unless stated otherwise. All lifespan assays were performed at 20 °C unless stated otherwise. Kaplan–Meier survival curves were plotted and log-rank statistics were calculated as described above.

For lifespans under reduced dietary conditions, 120 adult worms were transferred on a single plate and were moved only once, on day 8, to a fresh plate.

### Paraquat resistance of worms

Four-day old hermaphrodites were transferred to fresh NGM plates containing 20 mM Paraquat seeded with OP50. For experiments with *nsun-5* RNAi, worms were synchronized on RNAi plates and then transferred to OP50-seeded paraquat plates as above. Three days later, dead worms were counted. Data of at least three biological replicates were analysed with two-sided Student’s *t*-test implemented in GraphPad Prism version 5.00 for Windows (GraphPad Software).

### Size measurement of worms

Age-synchronous animals were grown at 20 °C on OP50 as described above. Three-day old hermaphrodites were placed on 2% agar pads and one drop of 1 mM levamisol (Sigma) was added to paralyse the worms. The length of the body in μm was measured in bright-field mode using a DMI6000 B inverted microscope (Leica Microsystems) equipped with a DFC360FX camera (Leica Microsystems). Results were analysed using two-sided Student’s *t*-test as above.

### Swimming locomotion analysis of worms

On day 1 and day 14 of adulthood, 20 single worms per sample were picked off agar plates seeded with either OP50 or the respective RNAi bacteria and transferred to 1 ml M9 buffer in a 24-well plate. After a 10- to 30-s recovery period, the number of body bends of the mid-body in 30 s was counted using a stereomicroscope. Only animals moving away after a gentle prod with a platinum wire were used for the assay^41^. Results were analysed using two-sided Student’s *t*-test as above.

### Pharyngeal pumping assays

Age-synchronous animals were grown at 20 °C on OP50 as described above. The number of contractions in the terminal bulb of the pharynx was measured on day 3 of adulthood. For each strain, 10–15 different animals were scored during a 60-s trial and compared using two-sided Student’s *t*-test as above.

### Yeast strains

Within this study, we used BY4741 (haploid, mating type a) as the wt strain. The *rcm1* (YNL022c) deletion strain (in BY4741, haploid, mating type a) was obtained from EuroSCARF and the deletion was verified by PCR. CEN.PK1170-1C (MATa ura3-52 trp1-289 leu2-3,112 his3▵1 ▵▵rDNA::pNOY455 [HIS3]+pPK655 (rDNA C2278G)) and CEN.PK968 (MATa ura3-52 trp1-289 leu2-3,112 his3▵1 ▵▵rDNA::pNOY455 [HIS3]+pPK622 (rDNA wt)) were provided by Karl-Dieter Entian^28^.

### Chronological lifespan

Chronological lifespan assays were performed using standard procedures^42^ with minor modifications: stationary cultures in 10 ml YPD medium (30 h after inoculation) were transferred to 10 ml sterile reverse osmosis-water and the water was replaced every 2 to 3 days. Cultures were kept in centrifuge tubes (Falcon) at 25 °C and shaking with 180 r.p.m. When water was exchanged, a small defined aliquot of the resuspended culture was streaked out on agar-solidified YPD-plates, incubated at 30 °C for 2 days and colonies were counted. The lifespan experiment reported was conducted three times, each with three technical replicates.

Chronological lifespan assays in SC medium were performed at 25 °C in 250 ml Erlenmeyer flasks. SC medium (25 ml) was inoculated to an OD of 0.1 with an overnight culture grown in SC medium. When the maximal cell density was reached (usually after 3–5 days), this day was recorded as day 0 and survival plating was performed as above every 2–4 days.

### Determination of oxidant- and heat shock sensitivity by serial dilution

Plate tests for testing the sensitivity to oxidants were performed by spotting overnight cultures of yeast cells onto SC-glucose plates containing increasing concentrations of H_2_O_2_ (0.4–2.4 mM) (ref. 43). Cells were grown to stationary phase in liquid SC-glucose, serially diluted to cell counts of 10^8^–10^3^ cells ml^−1^ and 10 μl aliquots of each dilution were spotted onto the appropriate plates to give a final cell number of 10^6^–10^1^. Stress sensitivity was determined by comparison of growth of *rcm1* mutant with that of the wt strain after incubation at 28 °C for 48 h or 7 days. One representative trial of three biological replicates is shown.

### Growth curve analysis for determining the sensitivity to oxidants

Growth curve analyses were performed in liquid SC medium in Erlenmeyer flasks. Cultures were prepared from overnight cultures by inoculating to an OD600 of 0.1. Different amounts of H_2_O_2_ (0.4–2.4 mM) were added and cells were incubated at 28 °C on a gyratory shaker. After defined time intervals, 1 ml samples were taken and measured at 600 nm with SC medium as blank.

### Replicative lifespan

Replicative lifespan assays were performed in SC at 28 °C for 30 cells per plate and two plates for each strain. Virgin cells that never budded were replaced. At least 50 cells per strain were examined. Lifespans were determined by counting all subsequent daughter cells generated, which were removed via micromanipulation^7^,^44^. Kaplan–Meier survival curves were plotted and log-rank statistics were calculated as above.

### Bisulfite sequencing

Bisulfite sequencing of RNA was performed as previously described^45^,^46^ with modifications. Primer sequences are provided in Supplementary Table 3. Total RNA or RNA isolated from polysome or monosome fractions was purified using Trizol (Life Technologies), DNAse I (Thermo Scientific) digested for 1 h, phenol–chloroform extracted, isopropanol precipitated and dissolved in 20 μl water. One μg RNA in 20 μl water was mixed with 42.5 μl bisulfite solution (Epitect Kit, Qiagen) and 17.5 μl DNA protect buffer (Epitect Kit, Qiagen) in a thin wall 100 μl PCR tube. In a thermo cycler, the following programme was run overnight: 70 °C for 10 min and 60 °C for 60 min for 6 cycles, 15 °C until further processing. RNA was desalted using a Micro Bio-Spin 6 SSC column (Biorad), mixed with 80 μl 1 M Tris-Cl (pH 9.0) buffer and incubated at 37 °C for 1 h. After isopropanol precipitation, 400 ng RNA was reverse transcribed using Super Script III reverse transcriptase (Life Technologies) and gene-specific reverse primers (Supplementary Table 3) for 50 min following the manufacturer’s instructions. Five μl complementary DNA (cDNA) was subjected to the following touchdown-PCR programme in a 25 μl reaction with KAPA2G Robust Polymerase and Buffer A (Kapa Biosystems):


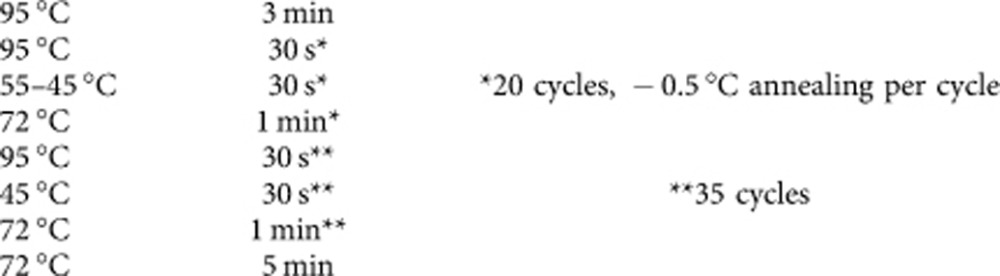


PCR products were gel purified using PCR- and Gel Extraction Kit (Favorgen) and eluded in 30 μl. Five μl was subjected to a second round of PCR as above using barcoded primers and only 30 cycles in the second cycling step (**).

Alternatively, the EZ RNA Methylation Kit (Zymo Research) was used for bisulfite treatment and purification of converted RNA according to the instructions provided by the manufacturer. Thereby, better reproducibility and higher conversion rates were obtained. Reverse transcription and PCR were done as described above.

For *C. elegans*, ~200 whole young adult hermaphrodites were washed 3 × in PBS and lysed directly in Trizol by 30 cycles of sonication (30 s on, 30 s off) in a Bioruptor (Diagenode). RNA isolation and bisulfite treatment were performed as described above.

Up to 24 barcoded samples were pooled to equal molarity and subjected to IonTorrent sequencing using a 314 Torrent Sequencing Chip Kit (Life Technologies).

Only reads with a completely sequenced bar code were considered for the following steps. First, all reads were sorted by their bar codes and bar codes were subsequently removed. Next, the reads were aligned to the reference sequences for *S. cerevisiae* 25S rRNA (J01355.1, position 2317–2438) and *C. elegans* 28S rRNA (NR_000055.1) using the Needleman–Wunsch algorithm, which finds the optimal global alignment for the given parameters^47^. Each sequence position was scored by following parameters:


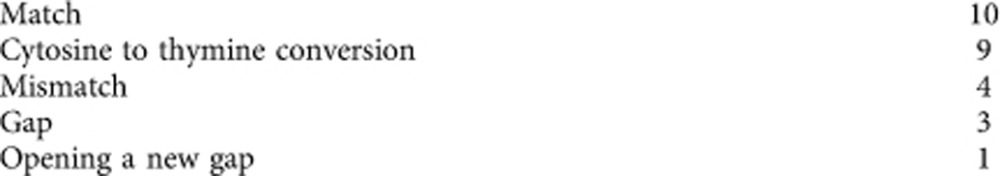


Before each alignment, the respective longer sequence (either the query- or the reference sequence) was trimmed, as is necessary for global alignments. In the next step, the aligned reads were filtered for sequences having less than 15% insertions or 15% deletions, or less than 20% insertions plus deletions. Sequences not fulfilling these requirements were removed. In the remaining sequences, the number of cytosines and thymines was counted at those positions, where the reference sequence had a cytosine. Furthermore, the overall rate of unconverted cytosines of each sample was determined by calculating the percentage of unconverted cytosines at each individual unmethylated C-position, and then taking the average overall positions. All C-positions except C2278 of 25S rRNA were considered as unmethylated.

### Ribosome gradients

Ribosome gradients were repeated five times with qualitatively and quantitatively similar results. wt and *rcm1*-knockout yeast were grown overnight in 3 ml SC medium at 28 °C with shaking. On the next day, 100 ml SC were inoculated with the overnight culture to an OD600 of 0.003 and grown at 28 °C with shaking until the OD600 reached 0.5–0.6. Then 0.4 mM H_2_O_2_ (only for stress conditions) and after 15 or 75 min, 125 μg ml^−1^ cycloheximide (Sigma) were added and the cells were incubated at the same temperature with shaking for further 15 min.

Later, the flasks were placed on ice for 15 min with shaking from time to time. After pelleting the cells at 3,500 r.p.m. and 4 °C for 5 min, the pellets were washed two times with 2 ml cooled TMNSH buffer (10 mM Tris pH 7.4, 50 mM NH_4_Cl, 10 mM MgCl_2_, 12.5 mM 2-mercaptoethanol) and resuspended in 1 ml of lysis buffer (10 mM Tris pH 7.4, 50 mM NH_4_Cl, 10 mM MgCl_2_, 12.5 mM 2-mercaptoethanol, 50 mM KCl, 1 mM PMSF (phenylmethyl sulphonyl fluoride), 125 μg ml^−1^ cycloheximid). Lysis was performed by transferring the suspensions to glass tubes and vortexing with glass beads (0.4–0.6 mm diameter) eight times for 30 s, with at least 30 s recovery on ice between each cycle. Afterwards, the crude lysates were centrifuged at 4,000 r.p.m. at 4 °C for 10 min to remove insoluble debris. The supernatants were transferred to fresh tubes and were supplemented with 100 μl Triton-X-100 (10% v/v). After centrifugation at full speed in a tabletop centrifuge at 4 °C for 10 min, the supernatants were carefully transferred to fresh vials and the OD260 was recorded for quantification. A volume equalling 15 OD260 was loaded onto a 10-ml linear 7–52% sucrose gradient in TMNSH buffer containing 50 mM KCl and centrifuged at 37,000 r.p.m. at 4 °C for 3 h. The profiles were recorded from the bottom of the tubes with an ultraviolet detector set to 254 nm.

### Luciferase PTC reporter assay

For every experimental setup, the wt and *rcm1* deletion strains were co-transformed with the Renilla luciferase (REN) reporter plasmid pLM161 and firefly luciferase (FF) reporters with or without a PTC^20^. This generated co-expressed REN/FF and REN/FFPTC reporter pairs in each strain. The luciferase assays were performed with the Dual-Luciferase Reporter Assay System and the reagents were prepared as indicated by the supplier (Promega Inc). Yeast cells were grown to exponential phase at 28 °C in SC medium. After harvesting, cell concentration was determined using a CASY Model TT cell counter (Roche Diagnostics) to analyse an equal amount of 1 × 10^7^ cells for each experiment. After centrifugation of the cell suspension, the resulting supernatant was removed and cells were lysed by dissolving in 1 ml of 1 × passive lysis buffer for 30 min at room temperature. After centrifugation to remove cell debris, 20 μl of the lysate was transferred into a black and white 96-multiwell isoplate (Perkin Elmer) and luminescence measurements were performed with the Glomax Multi Luminometer (Promega). For the FF and the REN reactions, 50 μl of LarII and 50 μl Stop and Glo, respectively, were automatically injected into the lysate. After a lag time of 2 s on substrate addition, luminescence signals were integrated for 10 s. Measurements were performed using two biological replicates each in triplicate to give a sum of six recordings for the respective REN and FF luminescence reporter pairs.

### Isolation of yeast 80S ribosomes and structural probing

wt and *rcm1*-knockout (*rcm1Δ*) yeast cells were grown in SC medium at 30 °C to a final OD600 of 0.6–0.8. Then 0.4 mM H_2_O_2_ was added for 30 or 90 min and cells were collected at 6,000*g*. The 80S ribosomes from unstressed and stressed cells were isolated as described previously^48^. Before primer extension analysis, 10 pmol of ribosomes were incubated with buffer (20 mM HEPES/KOH pH 7.6, 6 mM MgAc_2_, 150 mM NH_4_Cl, 2 mM spermidine, 0.05 mM spermine, 4 mM beta-mercaptoethanol) for 10 min at 37 °C. Chemical probing with DMS at 37 °C for 15 min was performed as described^49^. To monitor the DMS-reactive sites in domain IV of 25S rRNAs, primer extension analysis (primers used: 5′-GTTCCCTTGGCTGTGGTTTC-3′; 5′-TCTCGTTAATCCATTCATG-3′) was performed as previously described^50^. Primer extension products were separated on 8% denaturing polyacrylamide gels and visualized by using a PhosphorImager (FLA-3000; Fuji Photo Film) and quantified with the densitometric programme Aida Image Analyzer.

### Translational profiling and microarray analysis

Polysome profiling and stress treatment was performed as described above from two biological replicates on two different days. Fractions were collected and 300 μl whole-cell lysate or pooled polysome fractions were mixed with 900 μl Trizol LS (Life Technologies). RNA extraction was performed following the manufacturer’s instructions. A common reference pool was made by pooling same amounts of all samples. Samples were labelled with Cy3 and the common reference pool with Cy5 using the Low Input Quick Amp Labeling Kit Two Color (Agilent) following the manufactures instructions. Hybridization to Yeast (V2) 8 × 15 K Gene Expression Microarrays (Agilent) and washing were performed following the manufactures instructions. Slides were scanned with the two laser Agilent microarray scanner (G2565A). For raw data generation, Agilent feature extraction software (version 7.5) was used. Raw data were processed into R/Bioconductor. Intensities were log2 transformed, backgrounds were subtracted using normexp and intensities were normalized within arrays by using the Loess method and between arrays by using Aquantile. Adjustment for paired samples, which resulted from using the same lysates for total RNA isolation and polysome purification, was performed using ‘duplicateCorrelation’.

Ti-values were calculated using the limma package^51^ by forming the contrasts between the translatome and the respective transcriptome for wt and *rcm1Δ* samples with 0, 30 and 90 min stress. Limma was applied likewise for comparing different samples just within the transcriptome or translatome. *P* values resulting from moderated *t*-tests in limma were adjusted for multiple comparisons using the method by Benjamini Hochberg. Genes with a fold change of larger than two and *P*<0.05 were considered to be differentially regulated.

Gene set enrichment analysis (GSEA)^52^ was performed in R by generating gene lists of all genes ranked by their log2 fold change for each of the Ti-calculations, as well as for the comparisons within the transcriptome and translatome. Furthermore, a list of yeast genes and their respective gene ontology (GO) terms linked with ‘biological process’ was constructed in R using the GO.db and GSEABase packages. GSEA was then conducted in GSEA v2.1.0 (Broad Institute) using the following parameters: 1,000 permutations, maximum size of gene set: 1,000 and minimum size of gene set: 5.

### RT–qPCRs of ribosome gradient fractions

Polysome profiling and RNA isolation were performed as described above from three biological replicates on three different days. Isolated RNA was reverse transcribed into cDNA by DyNAmo cDNA Synthesis Kit (Finnzymes). Target gene expression levels were measured by SYBR-green based qPCR using the 5x HOT FIREPol EvaGreen qPCR Mix Plus (Medibena) with gene-specific primers (see Supplementary Table 3). Expression levels of target genes were normalized to *TDH3*, *TUB1* and *ACT1* and averaged.

## Additional information

**Accession codes:** RNA bisulfite sequencing data have been deposited in NCBI's Gene Expression Omnibus (GEO) under accession code GSE63113. Microarray data have been deposited in NCBI's Gene Expression Omnibus (GEO) under accession code GSE63030.

**How to cite this article:** Schosserer, M. *et al.* Methylation of ribosomal RNA by NSUN5 is a conserved mechanism modulating organismal lifespanlifespan. *Nat. Commun.* 6:6158 doi: 10.1038/ncomms7158 (2015).

## Supplementary Material

Supplementary Figures and Supplementary Tables Supplementary Figures 1-9 and Supplementary Tables 1-3.

Supplementary Data 1 Regulated single genes in translatome versus transcriptome. Summary tables of single genes, which were significantly up- or down-regulated (p = 0.05, multiple comparison adjusted t-test) in the transcriptome compared to the translatome in wild-type and rcm1 knock-out yeast with and without oxidative stress.

Supplementary Data 2 Overlap of regulated genes under different conditions. Table summarizing single genes, which were commonly regulated under different oxidative stress conditions and genotypes (see corresponding Figure 7b-e).

Supplementary Data 3 Regulated gene sets in translatome versus transcriptome. Summary tables of gene sets, which were significantly (FDR < 25%, gene set enrichment analysis) upor down-regulated in the transcriptome compared to the translatome in wild-type and *rcm1* knockout yeast with and without oxidative stress.
