# Corrigendum: Methylation of ribosomal RNA by NSUN5 is a conserved mechanism modulating organismal lifespan

**DOI:** 10.1038/ncomms11530

**Published:** 2016-05-11

**Authors:** Markus Schosserer, Nadege Minois, Tina B. Angerer, Manuela Amring, Hanna Dellago, Eva Harreither, Alfonso Calle-Perez, Andreas Pircher, Matthias Peter Gerstl, Sigrid Pfeifenberger, Clemens Brandl, Markus Sonntagbauer, Albert Kriegner, Angela Linder, Andreas Weinhäusel, Thomas Mohr, Matthias Steiger, Diethard Mattanovich, Mark Rinnerthaler, Thomas Karl, Sunny Sharma, Karl-Dieter Entian, Martin Kos, Michael Breitenbach, Iain B. H. Wilson, Norbert Polacek, Regina Grillari-Voglauer, Lore Breitenbach-Koller, Johannes Grillari

Nature Communications 6: Article number: 6158 10.1038/ncomms7158 (2015); Published: 01302015; Updated: 05112016

This Article contains an error in the numbering of the *C. elegans* 25S rRNA site methylated by *nsun-5*, which was incorrectly given as C3381. The correct methylation site is C2381. The correct version of Fig. 4 and its legend is depicted below. In addition, in the Discussion section of this Article, the sentence ‘By applying the same method to *C. elegans*, we could prove that C3381, which corresponds to yeast C2278, is indeed methylated by *nsun-5* (Fig. 4e)' should read ‘By applying the same method to *C. elegans*, we could prove that C2381, which corresponds to yeast C2278, is indeed methylated by *nsun-5* (Fig. 4e).'

[Fig i1]

## Figures and Tables

**Figure i1:**
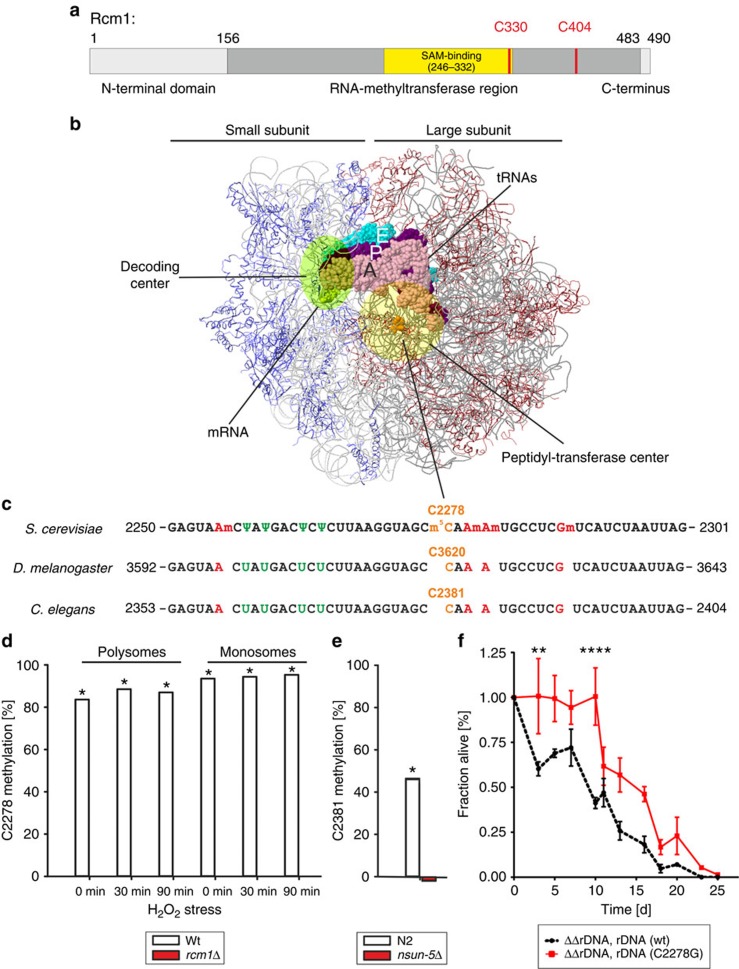
**Figure 4 | The conserved m^5^C-rRNA methyltransferase activity of Rcm1 mediates lifespan extension.** (**a**) Rcm1 is a protein of 490 amino acids harbouring an RNA methyltransferase domain with two highly conserved cysteines, C330 and C404, which are predicted to participate in the catalysis of methyl transfer. (**b**) Model of the yeast ribosome with functional elements. The large and small ribosomal subunits, RPs of the SSU in blue and of the LSU in red, are shown assembled on the mRNA, the tRNAs in the aminoacyl site (A, pink), in the peptidyl site (P, magenta) and in the exit site (E, cyan) are depicted, and cytosine 2278 (C2278) of the 25S rRNA in vicinity of the peptidyltransferase centre is highlighted in orange. (**c**) 25S rRNA sequence tract, nucleotide 2251 to 2300, harbours a series of highly conserved modified nucleotides, pseudouridinylations in green and guanine and adenine base methylations in red, and the single m^5^C-methylation in orange. This region is 100% conserved from *S. cerevisiae* to *C. elegans* and *D. melanogaster*. (**d**,**e**) Bisulfite sequencing of wt yeast cells detects C2278 methylation in rRNA isolated from ribosomal fractions independent of oxidative stress. Deletion of rcm1 resulted in a complete lack of cysteine C2278 methylation under all conditions tested (**d**). Bisulfite sequencing of N2 wt *C. elegans* confirms conservation of C2381 m^5^C-methylation, while deletion of nsun-5 resulted in a complete lack of this modification (**e**). Displayed values represent fraction of C2278/C2381 methylation minus average fraction of unconverted Cytosines except C2278/C2381 as unspecific background. Grubbs' Test at α=0.01 was performed on all Cytosines in the sample and asterisk * marks samples with C2278/C2381 identified as significant outlier. (**f**) Deletion of ribosomal DNA in haploid *S. cerevisiae* and rescue with unmethylatable rDNA (C2278G) on a plasmid increases chronological lifespan in SC compared with wt rDNA (wt). Error bars represent s.e.m. of three biological replicates (multiple comparison adjusted two-way ANOVA with Sidak post test, α=0.05, **P<0.01, ****P<0.0001).

