# Supplementary material for: Methylation of ribosomal RNA by NSUN5 is a conserved mechanism modulating organismal lifespan
Source: Nat Commun. 2015 Jan 30;6:6158. doi: 10.1038/ncomms7158 (PMC4317494; doi:10.1038/ncomms7158)
Supplement: Supplementary Figures and Supplementary Tables — Supplementary Figures 1-9 and Supplementary Tables 1-3. [file ncomms7158-s1.pdf]

a

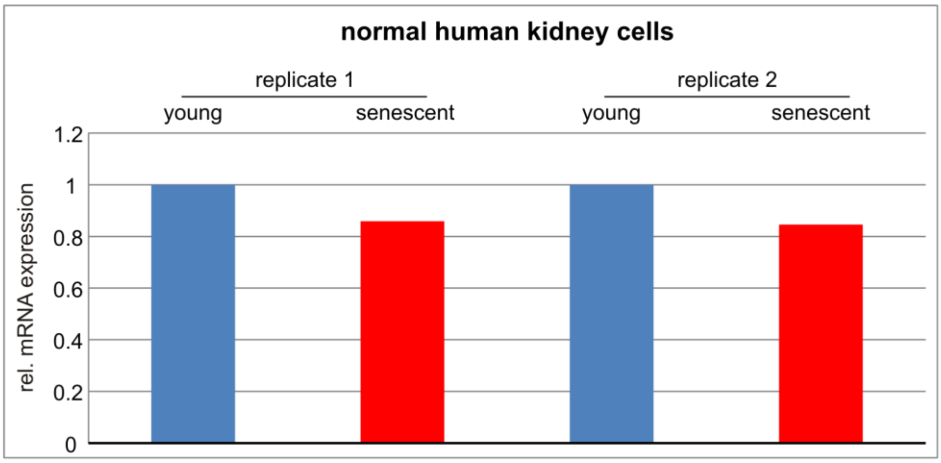

b

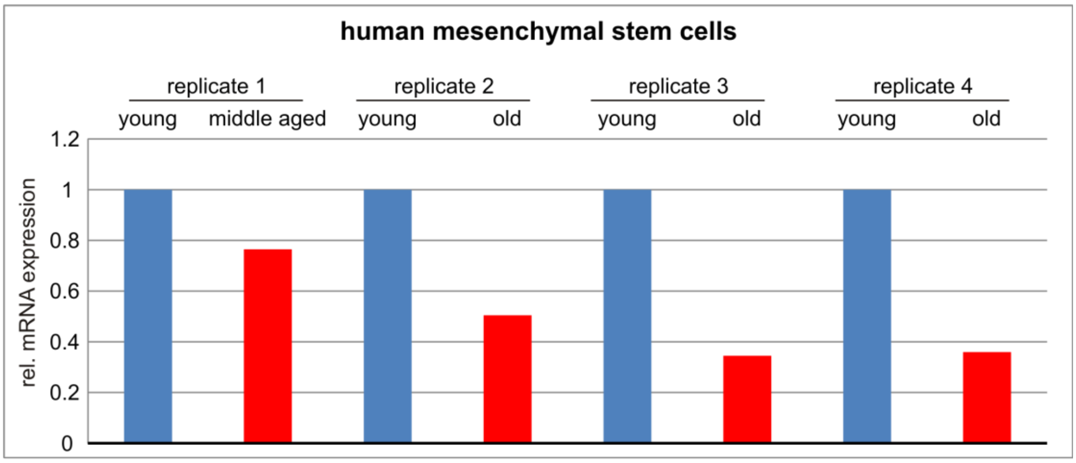

c

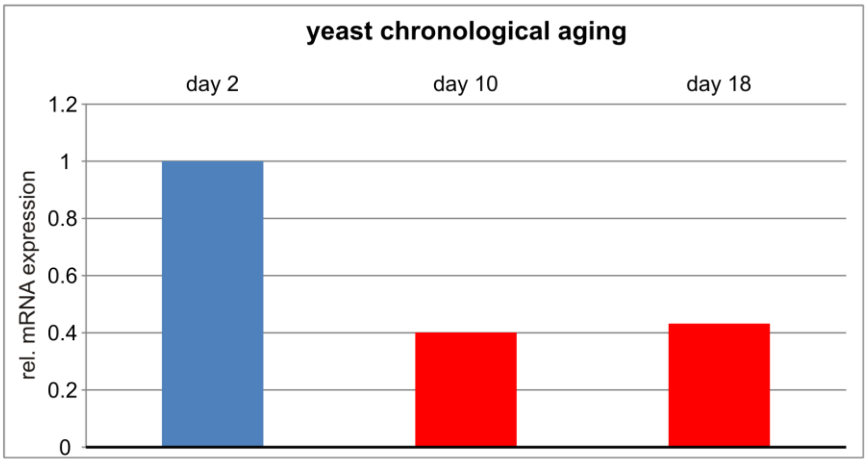

Supplementary Figure 1 | NSUN5/*RCM1* mRNA is less abundant in aged cells. Gisao.db database derived mRNA expression values of young and replicative-senescent normal human kidney cells from two different donors **(a)**, of mesenchymal stem cells from young, middle-aged and old donors **(b)** and of chronologically aged yeast **(c)**. For human cells (**a**,**b**) the mean expression of two probesets is shown. For yeast **(c)**, only one probeset for *RCM1* was available.

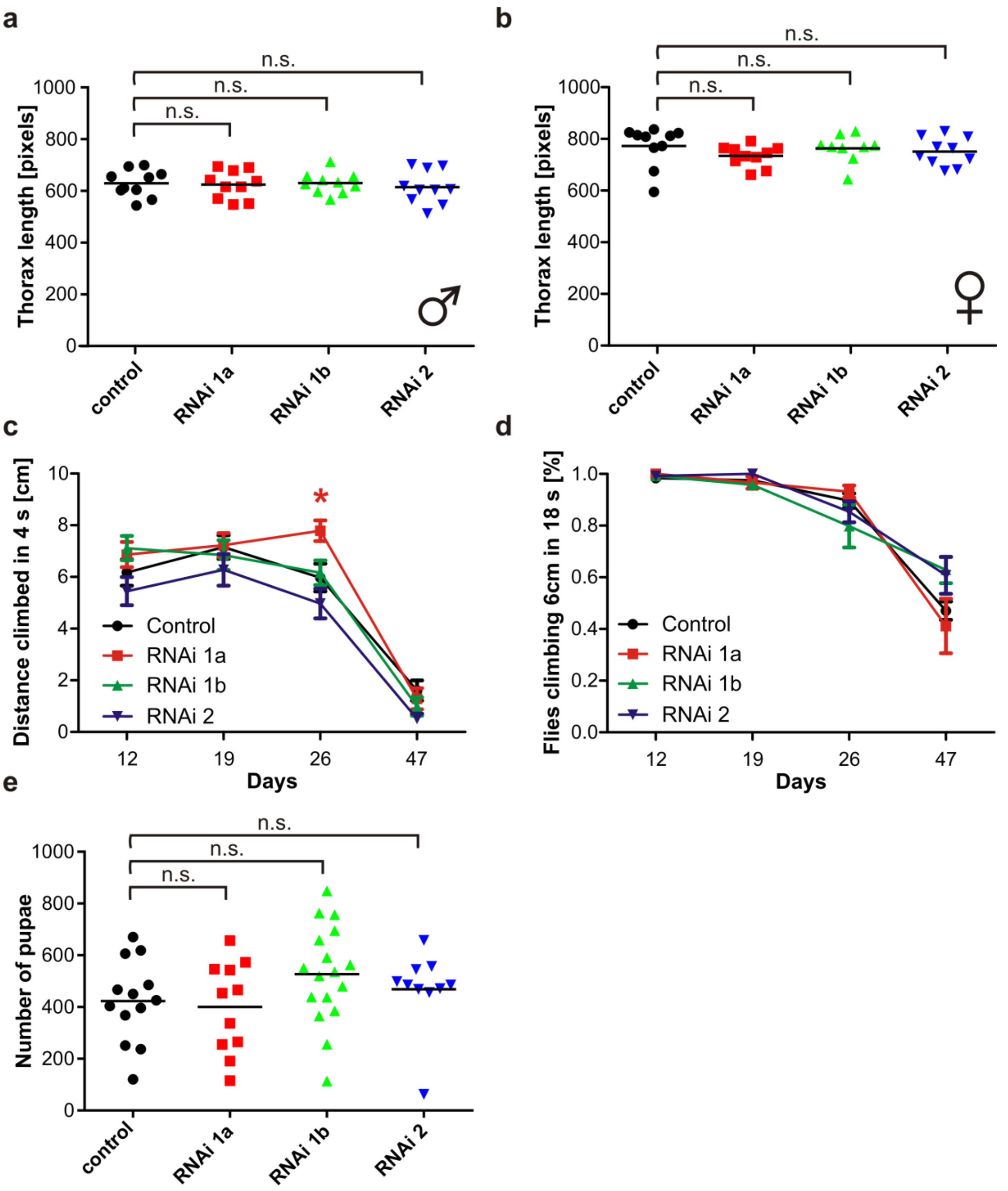

Supplementary Figure 2 | Phenotypic characterization of *dNsun5* knockdown flies. a-b, Mean thorax length in pixels of male **(a)** and female **(b)** control and three different *dNsun5* RNAi fly lines. Error bars represent S.E.M. N = 10, α = 0.05, One-way ANOVA with Dunnett's post test not significant for all conditions. **d-e,** Locomotion of control and three different *dNsun5* RNAi fly lines at different ages measured by climbed distance in 4 s per fly, N = 40 **(c)** or number of flies climbing 6 cm in 18 s, N = 4 **(d)**. Two-way ANOVA with Bonferroni post tests at α = 0.05 was not significant unless marked otherwise, *p < 0.05. **e,** Number of pupae produced over 4 weeks by control and three different *dNsun5* RNAi fly lines. Error bars represent S.E.M. N = 20, α = 0.05, one-way ANOVA with Dunnett's post test not significant.

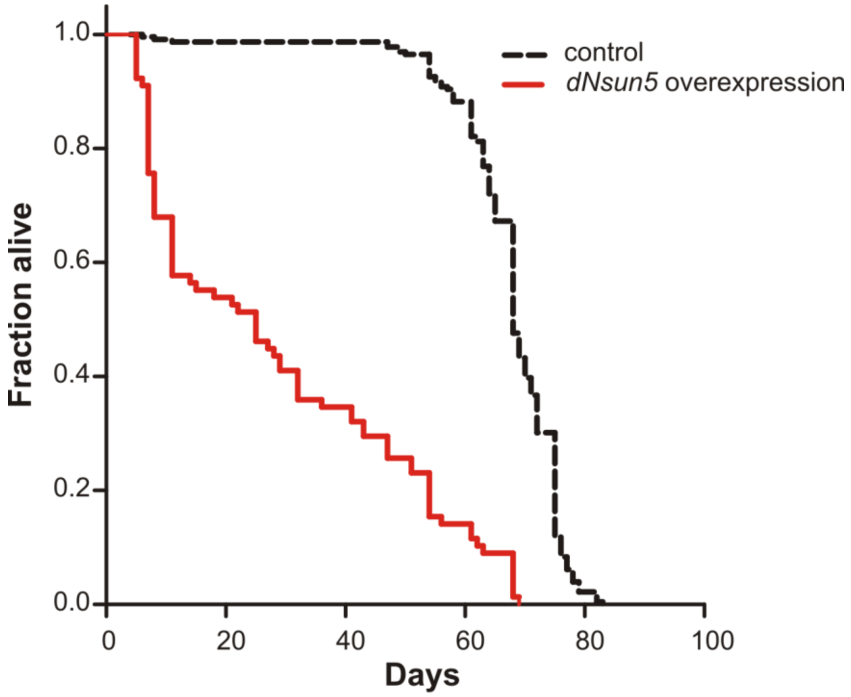

Supplementary Figure 3 | Overexpression of *dNsun5* shortens *Drosophila* lifespan. Kaplan-Meier survival curves of control and *dNsun5* overexpressing male *D. melanogaster* (average N per group = 78, log-rank p < 0.001).

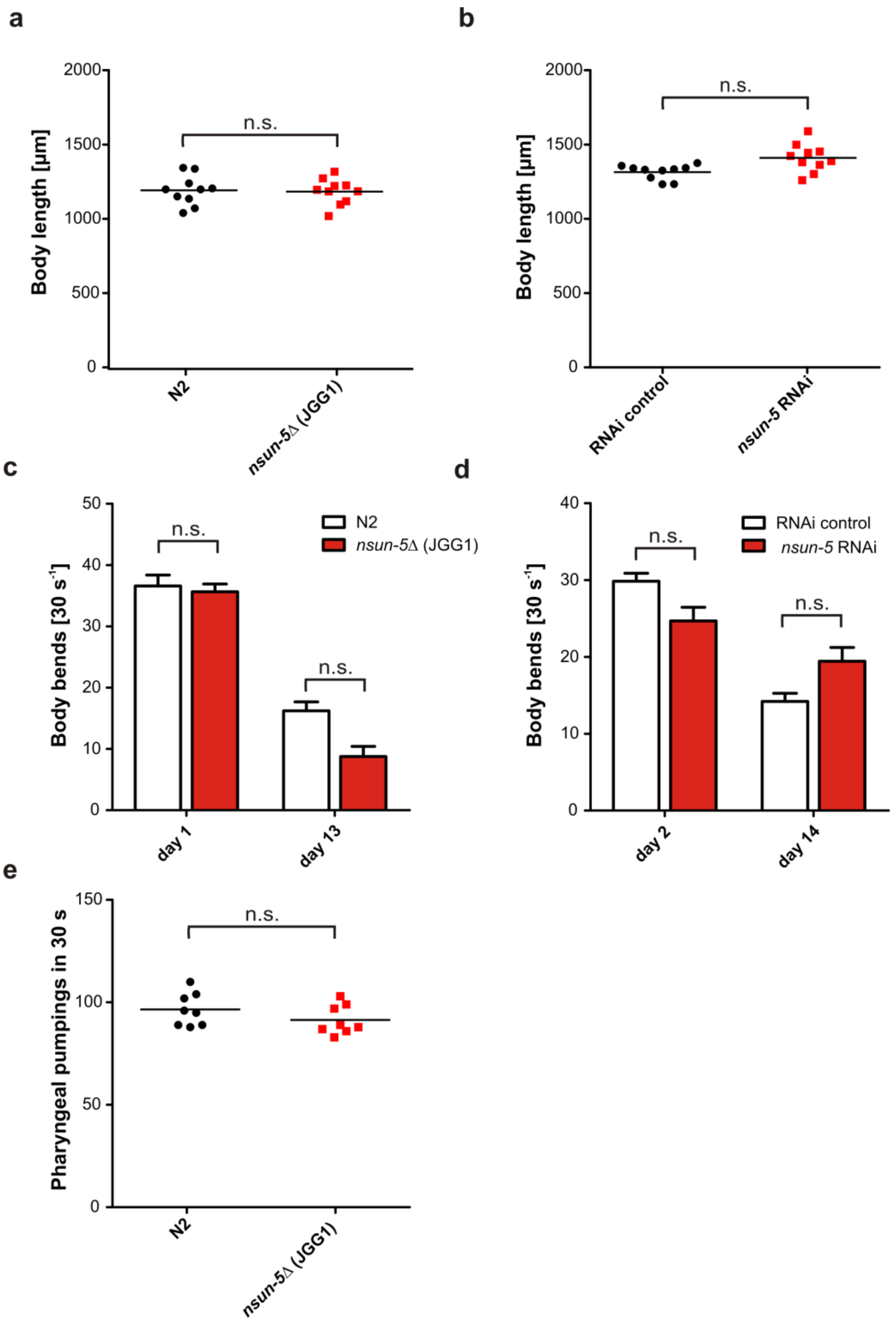

Supplementary Figure 4 | Phenotypic characterization of *nsun-5* knockout worms. a-b, Mean body length in µm of N2 vs. nsun-5 knockouts (JGG1) **(a)** or NL2099 subjected to control or *nsun-5* RNAi **(b)** hermaphrodites. Error bars represent S.E.M. N = 10, α = 0.05, two-sided Student's t-Test not significant. **c-d,** Locomotion activity at different timepoints of wild-type vs. *nsun-5* knockouts (JGG1) **(c)** or NL2099 subjected to control or *nsun-5* RNAi **(d)** hermaphrodites. Error bars represent S.E.M. N = 20, α = 0.05, two-sided Student's t-Test at different timepoints not significant. **e,** Pharyngeal pumping of wild-type vs. *nsun-5* knockout (JGG1) hermaphrodites. Error bars represent S.E.M. N = 20, α = 0.05, two-sided Student's t-Test not significant.

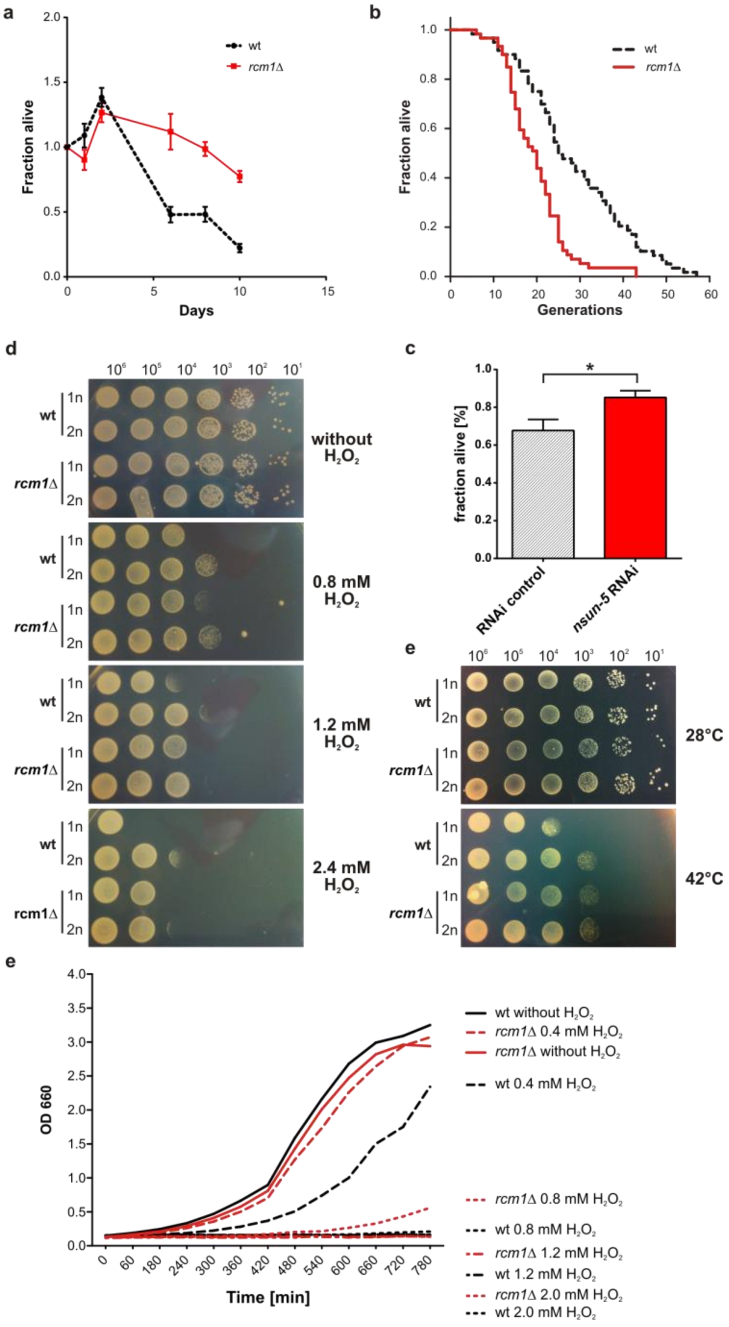

Supplementary Figure 5 | NSUN5 modulates stress resistance and chronological lifespan. a, Deletion of *rcm1* in haploid *S. cerevisiae* increases chronological lifespan in water. Error bars represent S.E.M. of 2-3 biological replicates. **b,** Replicative lifespan assay of haploid wild-type *S. cerevisiae* vs. a haploid *rcm1* knockout strain (N = 60, log-rank, $p < 0.001$). **c,** RNAi against *nsun-5* increases the paraquat resistance of N2 wild-type worms. Survival after 3 days of treatment with 20 mM paraquat are shown. N = 4 biological replicates with 30 worms each, $\alpha = 0.05$, two-tailed t-test $*p < 0.05$. Error bars represent S.E.M. **d, e,** Yeast serial dilution growth assays demonstrate increased resistance of haploid (1n) *rcm1Δ* cells compared to the haploid wild-type to H_2O_2 **(d)** and to heatshock **(e)**. Diploid (2n) heterozygously deleted *rcm1* cells compared to the diploid wild-type did not show increased survival, suggesting that a single copy of *RCM1* is sufficient. **f,** Liquid growth assay with increasing amounts of H_2O_2 in the medium shows increased survival of haploid *rcm1Δ* cells (red) compared to the wild-type (black).

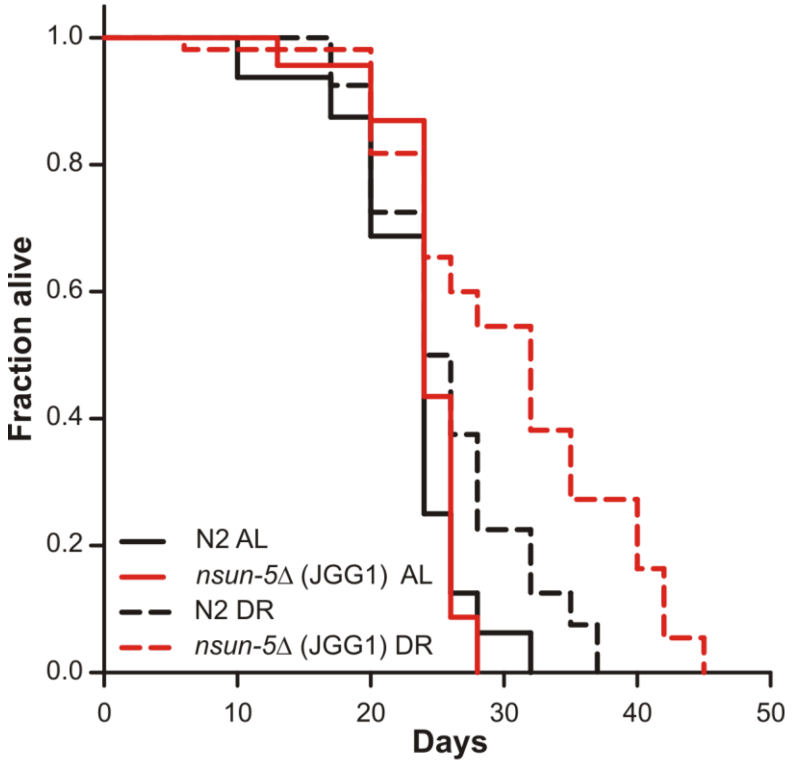

Supplementary Figure 6 | Lifespans of *nsun-5* mutants are only extended upon complete removal of food. Dietary restriction (DR) induced by complete removal of food during adulthood extends the lifespan of an *nsun-5* deletion mutant JGG1 (average N per group = 73, log-rank p = 0.03).

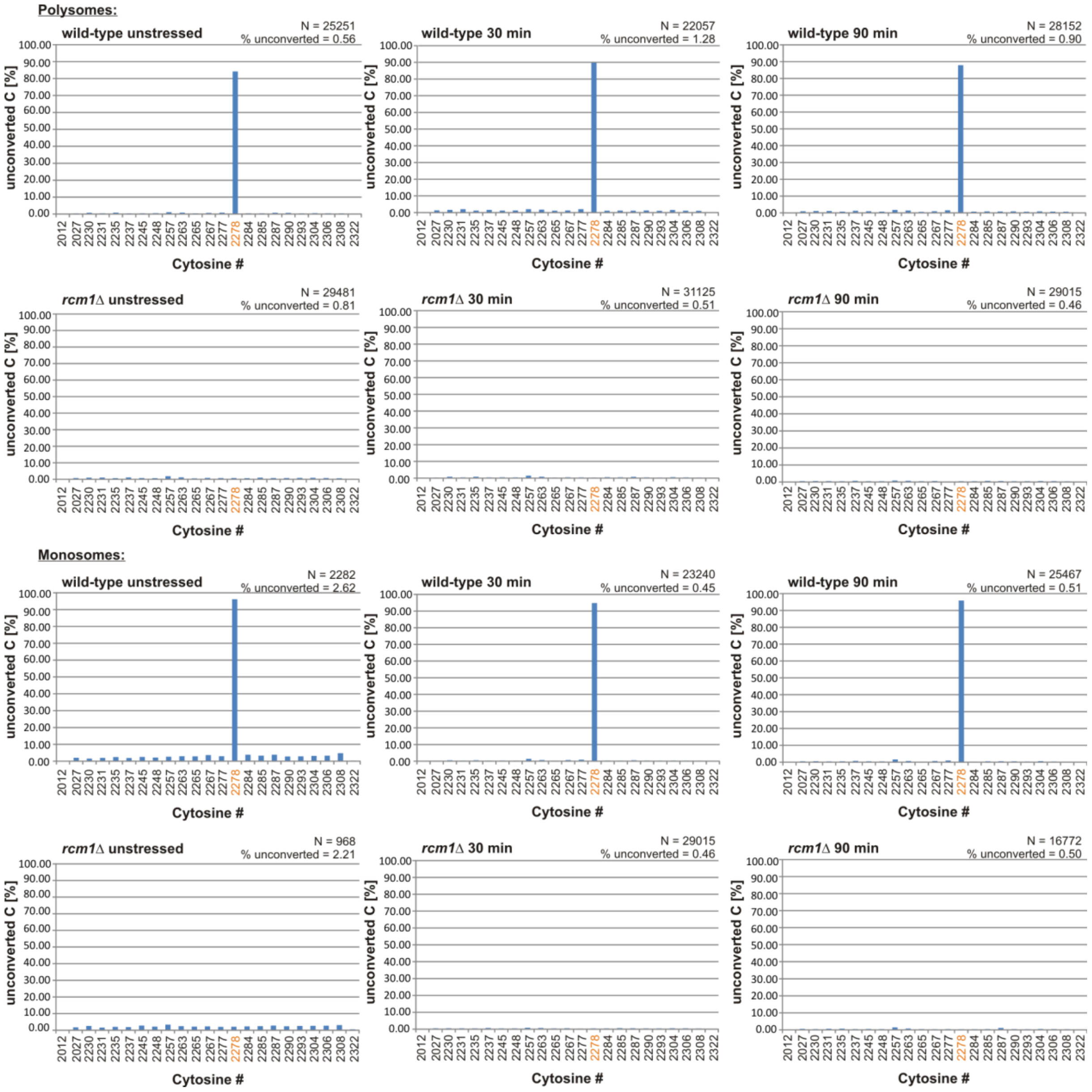

Supplementary Figure 7 | Bisulfite treatment demonstrates Rcm1-dependent methylation of C2278 in 25S rRNA. RNA isolated from ribosome fractions was bisulfite-sequenced. The percentage of unconverted (= C) to all (= C + T) cytosines at the indicated positions is represented by bars. C2278 is highlighted in orange. The number of full sequence reads is indicated (N), as well as the average percentage of unconverted cytosines at all positions except C2278.

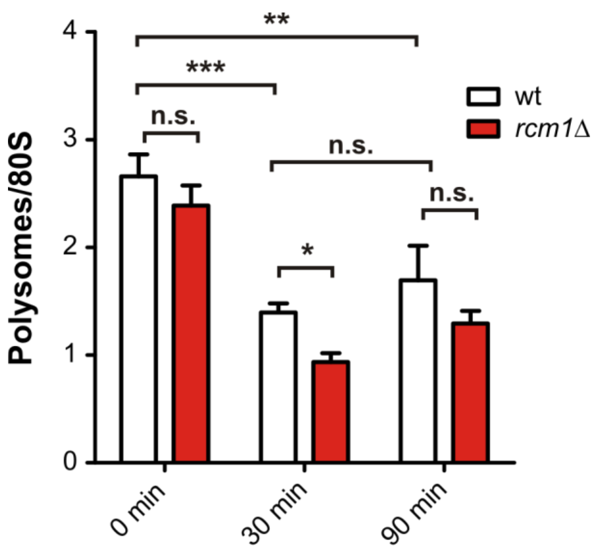

Supplementary Figure 8 | Quantification of polysomes to monosomes ratio of wt and *rcm1* knockout cells. The area under the curves of all recorded ribosome profiles was measured and the ratio of polysomes to monosomes was calculated. Error bars represent S.E.M. (N = 3-5, α = 0.05, Two-way ANOVA with Turkey's multiple comparisons test for testing effect of stress in wt, multiple comparison two-sided t-test using the Holm-Sidak method without assuming constant S.D. for comparing wild-type and *rcm1Δ* at different timepoints, ***$p < 0.001$, **$p < 0.01$, *$p < 0.05$).

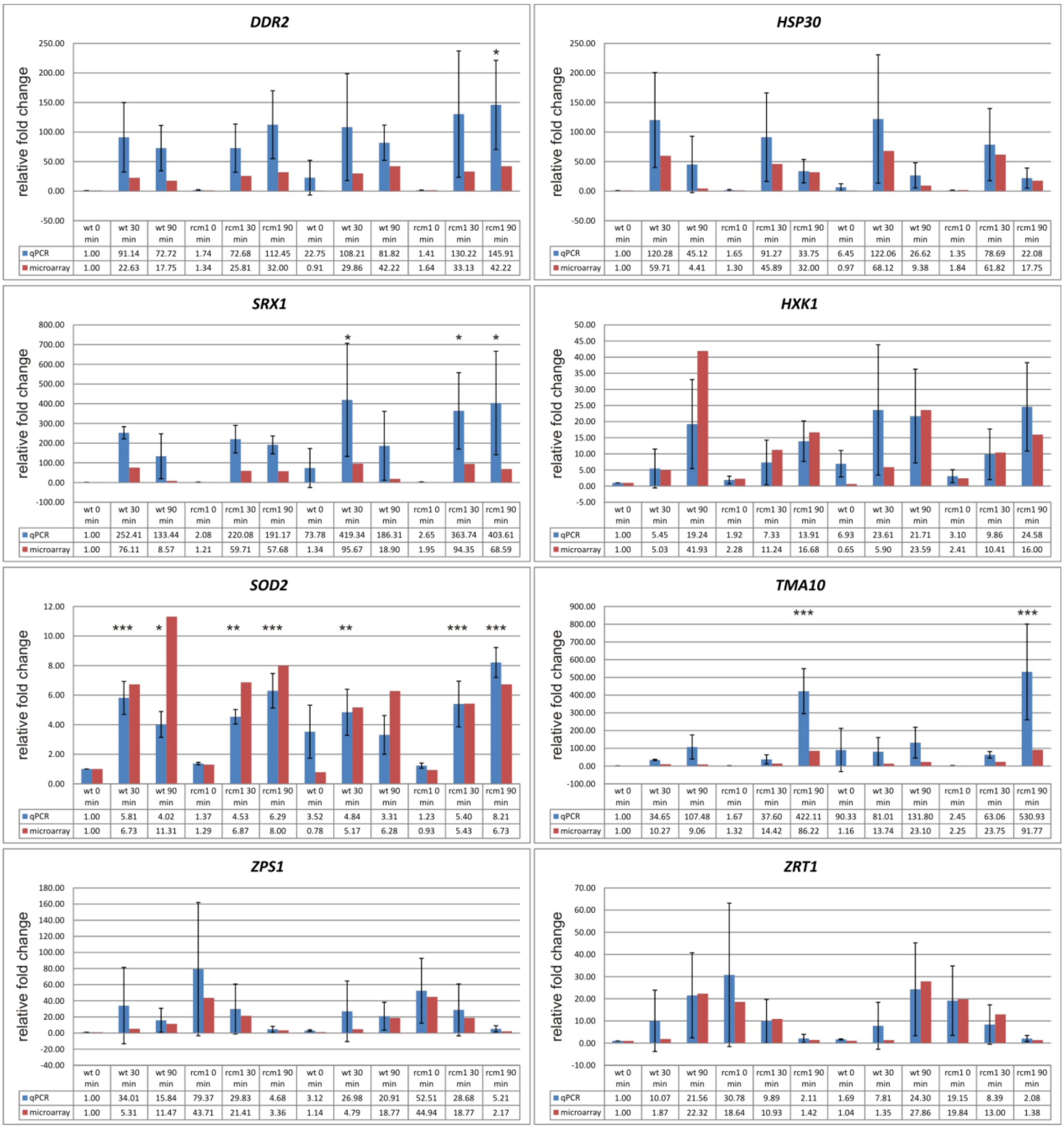

Supplementary Figure 9 | Verification of translational profiling microarrays by RT-qPCR. Relative expression levels of a subset of genes from the microarray (red) were validated by RT-qPCR (blue) on translatome and transcriptome level and show similar trends. Normalization was performed against *TDH-3*, *ACT-1* and *TUB-1*. The average target gene expression relative to all three housekeeping genes is depicted. Error bar represent S.D., N = 3 biological replicates, $\alpha = 0.05$, one-way ANOVA with Dunnett's post test, *$p < 0.05$, **$p < 0.005$, ***$p < 0.001$.

Strain	Mean lifespan +/- s.e.m. [days]	N	P	mean lifespan extension [%]
***dNsun5* RNAi (normal diet):**				
**<u>initial screen:</u>**				
**RNAi control**	55.9 +/- 0.8	147		
***dNsun5* RNAi 1a**	58.9 +/- 0.6	20	0.0077	5.37
***dNsun5* RNAi 1b**	59.1 +/- 2.0	20	0.0097	5.72
***dNsun5* RNAi 2**	53.6 +/- 1.2	20	0.0071	-4.11
**<u>replicate 1:</u>**				
**RNAi control**	50.8 +/- 1.2	168		
***dNsun5* RNAi 1a**	60.4 +/- 1.2	80	0.0002	18.90
***dNsun5* RNAi 1b**	59.0 +/- 1.6	80	< 0.0001	16.14
***dNsun5* RNAi 2**	61.4 +/- 1.3	82	< 0.0001	20.87
**<u>replicate 2:</u>**				
**RNAi control**	56.0 +/- 0.9	240		
***dNsun5* RNAi 1a**	59.8 +/- 1.3	74	0.0015	6.79
***dNsun5* RNAi 1b**	62.1 +/- 1.6	85	0.0010	10.89
***dNsun5* RNAi 2**	61.6 +/- 1.9	84	0.0002	10.00
***dNsun5* overexpression:**				
**<u>replicate 1 (25°C):</u>**				

**control**	67.7 +/- 0.7	229		
***dNsun5* contruct #1**	28.7 +/- 2.5	78	< 0,001	-57.61
***dNsun5* contruct #2**		embryonic lethal		

replicate 2 (20°C):

**control**	67.3 +/- 0.5	291		
***dNsun5* construct #1**	60.8 +/- 1.2	102	< 0,001	-9.66
***dNsun5* construct #2**		embryonic lethal		

***dNsun5* RNAi (sugar and yeast rich diet):**

replicate 1:

**RNAi control**	56.8 +/- 1.2	85		
***dNsun5* RNAi 1a**	50.2 +/- 1.3	93	0.00322	-11.62
***dNsun5* RNAi 1b**	60.2 +/- 1.0	89	0.140	5.99
***dNsun5* RNAi 2**	61.8 +/- 0.7	84	0.128	8.80

replicate 2:

**RNAi control**	61.9 +/- 1.0	73		
***dNsun5* RNAi 1a**	50.4 +/- 1.6	98	0.000153	-18.58
***dNsun5* RNAi 1b**	61.9 +/- 1.2	90	0.318	0.00
***dNsun5* RNAi 2**	63.9 +/- 0.9	91	0.0728	3.23

Supplementary Table 1 | Overview of *D. melanogaster* lifespans

Strain	Mean +/- s.e.m. days	Number of animals that died/total	*P*	mean lifespan extension [%]

NL2099 (reduced diet):

replicate1:

**RNAi control**	15.2 +/- 0.4	108/120		
***nsun-5* RNAi**	21.0 +/- 0.7	88/120	<0.001	38.16

replicate 2:

**RNAi control**	15.4 +/- 0.3	113/120		
***nsun-5* RNAi**	17.3 +/- 0.3	118/120	0.004	12.34

replicate 3:

**RNAi control**	15.2 +/- 0.3	116/120		
***nsun-5* RNAi**	17.5 +/- 0.3	103/120	<0.001	15.13

replicate 4:

**RNAi control**	14.6 +/- 0.3	116/120		
***nsun-5* RNAi**	18.2 +/ 0.2	114/120	<0.001	24.65

NL2099 (ad libidum fed):

replicate1:

**RNAi control**	23.5 +/- 0.4	103/120		
***nsun-5* RNAi**	23.8 +/- 0.3	104/120	0.552	1.28

replicate 2:

**RNAi control**	31.6 +/- 0.7	34/120		
***nsun-5* RNAi**	30.7 +/- 0.6	53/120	0.513	-2.85

N2 vs. JGG1 (normal diet):

replicate1:

**N2**	18.8 +/- 0.3	74/120		
**JGG1**	18.1 +/- 0.3	96/120	0.144	-3.72

replicate2:

**N2**	17.7 +/- 0.3	109/120		
**JGG1**	17.8 +/- 0.3	111/120	0.777	0.56

N2 vs. JGG1 (complete removal of food):

replicate1:

**N2**	25.9 +/- 0.9	40/73		
**JGG1**	30.7 +/- 2.0	19/74	0.033	18.53

replicate 2:

**N2**	11.2 +/- 3.0	6/47		
**JGG1**	15.6 +/- 2.3	9/48	0.145	39.29

DA1116 (*eat-2* mutation):

replicate 1:

**RNAi control**	23.7 +/- 0.8	55/120		
***nsun-5* RNAi**	27.4 +/- 0.9	51/120	<0.001	15.61

replicate 2:

**RNAi control**	25.4 +/- 0.6	90/120		
***nsun-5* RNAi**	28.0 +/- 0.6	93/120	<0.001	10.24

Supplementary Table 2 | Overview of *C. elegans* lifespans

qPCR primers:

DDR2_qPCR_sense	TCTGCCATCTCTGTCTTCGG
DDR2_qPCR_as	TCAAAAAGGCCAAAGCACCA
HSP30_qPCR_sense	GCTAATTGCAGTCAGCCGTG
HSP30_qPCR_as	TCTGCTTCAGGTTCGGGTTC
SRX1_qPCR_sense	CTCTAGAACAGGCTGAGGCG
SRX1_qPCR_as	CAGGTACATGCGGATTTGGC
HXK1_qPCR_sense	AATGTCGAAGGCCACGATGT
HXK1_qPCR_as	CCGTTGACACCAGTACCGAA
SOD2_qPCR_sense	CTCCCGCAAACGCAAGAAAA
SOD2_qPCR_as	CTCGTCCAGACTGCCAAACT
TMA10_qPCR_sense	TAACGGCAACTTTGGGGAGT
TMA10_qPCR_as	TGGGAGTTAGAGCCCCTTCT
ZPS1_qPCR_sense	ACCGCTGAAATCACCGCTTA
ZPS1_qPCR_as	TAGCTGCACACAAGCCATCA
ZRT1_qPCR_sense	ATGGACCCTGCTTATGGTGC
ZRT1_qPCR_as	TCGTGGGTATGGTCATGGGA
ACT1_qPCR_sense	TGAGAATCGATTTGGCCGGT
ACT1_qPCR_as	GATTGAGCAGCGGTTTGCAT
TDH3_qPCR_sense	TGCCATGGGGTTCTTCCAACG
TDH3_qPCR_as	TGGGGCGGTGGAAGATGGAG
TUB1_qPCR_sense	ACTGGTTCCGGTCTAGGGTC
TUB1_qPCR_as	TGCAAAGCTTGGTCTTGGGA

Bisulfite Sequencing primers:

SC-25S-rRNA_bisulfite_sense	TGGTTAGAAAGTGATGTT
SC-25S-rRNA_bisulfite_as	TCTCATTAATCCATTCA
CE-28S-rRNA_bisulfite_sense	TGAATGTTAAATTGTAGTAATT
CE-28S-rRNA_bisulfite_as	TCTCGTTAATCCATTCA

Barcode primers:

SC-25S-rRNA_torrent_code1f	CCATCTCATCCCTGCGTGTCTCCGACTCAGACGAGTGCGTTTTGAATGTTAAAGTGAA
SC-25S-rRNA_torrent_code1r	CCTCTCTATGGGCAGTCGGTGATACGAGTGCGTTCTCATTAATCCATTCA
SC-25S-rRNA_torrent_code2f	CCATCTCATCCCTGCGTGTCTCCGACTCAGACGCTCGACATTTGAATGTTAAAGTGAA
SC-25S-rRNA_torrent_code2r	CCTCTCTATGGGCAGTCGGTGATACGCTCGACATCTCATTAATCCATTCA
SC-25S-rRNA_torrent_code3f	CCATCTCATCCCTGCGTGTCTCCGACTCAGAGACGCACTCTTTGAATGTTAAAGTGAA
SC-25S-rRNA_torrent_code3r	CCTCTCTATGGGCAGTCGGTGATAGACGCACTCTCTCATTAATCCATTCA
SC-25S-rRNA_torrent_code4f	CCATCTCATCCCTGCGTGTCTCCGACTCAGAGCACTGTAGTTTGAATGTTAAAGTGAA

SC-25S-rRNA_torrent_code4r	CCTCTCTATGGGCAGTCGGTGATAGCACTGTAGTCTCATTAATCCATTCA
SC-25S-rRNA_torrent_code5f	CCATCTCATCCCTGCGTGTCTCCGACTCAGATCAGACACGTTTGAATGTTAAAGTGAA
SC-25S-rRNA_torrent_code5r	CCTCTCTATGGGCAGTCGGTGATATCAGACACGTCTCATTAATCCATTCA
SC-25S-rRNA_torrent_code6f	CCATCTCATCCCTGCGTGTCTCCGACTCAGATATCGCGAGTTTGAATGTTAAAGTGAA
SC-25S-rRNA_torrent_code6r	CCTCTCTATGGGCAGTCGGTGATATATCGCGAGTCTCATTAATCCATTCA
SC-25S-rRNA_torrent_code7f	CCATCTCATCCCTGCGTGTCTCCGACTCAGCGTGTCTCTATTTGAATGTTAAAGTGAA
SC-25S-rRNA_torrent_code7r	CCTCTCTATGGGCAGTCGGTGATCGTGTCTCTATCTCATTAATCCATTCA
SC-25S-rRNA_torrent_code8f	CCATCTCATCCCTGCGTGTCTCCGACTCAGCTCGCGTGTCTTTGAATGTTAAAGTGAA
SC-25S-rRNA_torrent_code8r	CCTCTCTATGGGCAGTCGGTGATCTCGCGTGTCTCTCATTAATCCATTCA
SC-25S-rRNA_torrent_code9f	CCATCTCATCCCTGCGTGTCTCCGACTCAGTCTCTATGCGTTTGAATGTTAAAGTGAA
SC-25S-rRNA_torrent_code9r	CCTCTCTATGGGCAGTCGGTGATTCTCTATGCGTCTCATTAATCCATTCA
SC-25S-rRNA_torrent_code10f	CCATCTCATCCCTGCGTGTCTCCGACTCAGTGATACGTCTTTGAATGTTAAAGTGAA
SC-25S-rRNA_torrent_code10r	CCTCTCTATGGGCAGTCGGTGATTGATACGTCTTCTCATTAATCCATTCA
SC-25S-rRNA_torrent_code11f	CCATCTCATCCCTGCGTGTCTCCGACTCAGATAGTAGTGTTTGAATGTTAAAGTGAA
SC-25S-rRNA_torrent_code11r	CCTCTCTATGGGCAGTCGGTGATATAGTAGTGTCTCATTAATCCATTCA
SC-25S-rRNA_torrent_code12f	CCATCTCATCCCTGCGTGTCTCCGACTCAGCGAGAGATATTTGAATGTTAAAGTGAA
SC-25S-rRNA_torrent_code12r	CCTCTCTATGGGCAGTCGGTGATCGAGAGATATCTCATTAATCCATTCA
SC-25S-rRNA_torrent_code13f	CCATCTCATCCCTGCGTGTCTCCGACTCAGATACGACGTATTTGAATGTTAAAGTGAA
SC-25S-rRNA_torrent_code14f	CCATCTCATCCCTGCGTGTCTCCGACTCAGTCACGTACTATTTGAATGTTAAAGTGAA
SC-25S-rRNA_torrent_code15f	CCATCTCATCCCTGCGTGTCTCCGACTCAGCGTCTAGTACTTTGAATGTTAAAGTGAA
SC-25S-rRNA_torrent_code16f	CCATCTCATCCCTGCGTGTCTCCGACTCAGTCTACGTAGCTTTGAATGTTAAAGTGAA
SC-25S-rRNA_torrent_code17f	CCATCTCATCCCTGCGTGTCTCCGACTCAGTGTACTACTTTTGAATGTTAAAGTGAA
SC-25S-rRNA_torrent_code18f	CCATCTCATCCCTGCGTGTCTCCGACTCAGACGACTACAGTTTGAATGTTAAAGTGAA
SC-25S-rRNA_torrent_code19f	CCATCTCATCCCTGCGTGTCTCCGACTCAGCGTAGACTAGTTTGAATGTTAAAGTGAA
SC-25S-rRNA_torrent_code20f	CCATCTCATCCCTGCGTGTCTCCGACTCAGTACGAGTATGTTTGAATGTTAAAGTGAA
SC-25S-rRNA_torrent_code21f	CCATCTCATCCCTGCGTGTCTCCGACTCAGTACTCTCGTGTTTGAATGTTAAAGTGAA
SC-25S-rRNA_torrent_code22f	CCATCTCATCCCTGCGTGTCTCCGACTCAGTAGAGACGAGTTTGAATGTTAAAGTGAA
SC-25S-rRNA_torrent_code23f	CCATCTCATCCCTGCGTGTCTCCGACTCAGTCGTCGCTCGTTTGAATGTTAAAGTGAA
SC-25S-rRNA_torrent_code24f	CCATCTCATCCCTGCGTGTCTCCGACTCAGACATACGCGTTTGAATGTTAAAGTGAA
SC-25S-rRNA_torrent_code13r	CCTCTCTATGGGCAGTCGGTGATATACGACGTATCTCATTAATCCATTCA
SC-25S-rRNA_torrent_code14r	CCTCTCTATGGGCAGTCGGTGATTCACGTACTATCTCATTAATCCATTCA
SC-25S-rRNA_torrent_code15r	CCTCTCTATGGGCAGTCGGTGATCGTCTAGTACTCTCATTAATCCATTCA
SC-25S-rRNA_torrent_code16r	CCTCTCTATGGGCAGTCGGTGATTCTACGTAGCTCTCATTAATCCATTCA
SC-25S-rRNA_torrent_code17r	CCTCTCTATGGGCAGTCGGTGATTGTACTACTTCTCATTAATCCATTCA
SC-25S-rRNA_torrent_code18r	CCTCTCTATGGGCAGTCGGTGATACGACTACAGTCTCATTAATCCATTCA
SC-25S-rRNA_torrent_code19r	CCTCTCTATGGGCAGTCGGTGATCGTAGACTAGTCTCATTAATCCATTCA
SC-25S-rRNA_torrent_code20r	CCTCTCTATGGGCAGTCGGTGATTACGAGTATGTCTCATTAATCCATTCA
SC-25S-rRNA_torrent_code21r	CCTCTCTATGGGCAGTCGGTGATTACTCTCGTGTCTCATTAATCCATTCA
SC-25S-rRNA_torrent_code22r	CCTCTCTATGGGCAGTCGGTGATTAGAGACGAGTCTCATTAATCCATTCA
SC-25S-rRNA_torrent_code23r	CCTCTCTATGGGCAGTCGGTGATTCGTCGCTCGTCTCATTAATCCATTCA
SC-25S-rRNA_torrent_code24r	CCTCTCTATGGGCAGTCGGTGATACGAGTGCGTTCTCATTAATCCATTCA
CE-28S-rRNA_torrent_code1f	CCATCTCATCCCTGCGTGTCTCCGACTCAGACGAGTGCGTTGAATGTTAAATTGTAGTAATT
CE-28S-rRNA_torrent_code2f	CCATCTCATCCCTGCGTGTCTCCGACTCAGACGCTCGACATGAATGTTAAATTGTAGTAATT
CE-28S-rRNA_torrent_code3f	CCATCTCATCCCTGCGTGTCTCCGACTCAGAGACGCACTCTGAATGTTAAATTGTAGTAATT
CE-28S-rRNA_torrent_code4f	CCATCTCATCCCTGCGTGTCTCCGACTCAGAGCACTGTAGTGAATGTTAAATTGTAGTAATT

CE-28S-rRNA_torrent_code5f	CCATCTCATCCCTGCGTGTCTCCGACTCAGATCAGACACGTGAATGTTAAATTGTAGTAATT
CE-28S-rRNA_torrent_code6f	CCATCTCATCCCTGCGTGTCTCCGACTCAGATATCGCGAGTGAATGTTAAATTGTAGTAATT
CE-28S-rRNA_torrent_code7f	CCATCTCATCCCTGCGTGTCTCCGACTCAGCGTGTCTCTATGAATGTTAAATTGTAGTAATT
CE-28S-rRNA_torrent_code1r	CCTCTCTATGGGCAGTCGGTGATACGAGTGCGTTCTCGTTAATCCATTCA
CE-28S-rRNA_torrent_code2r	CCTCTCTATGGGCAGTCGGTGATACGCTCGACATCTCGTTAATCCATTCA
CE-28S-rRNA_torrent_code3r	CCTCTCTATGGGCAGTCGGTGATAGACGCACTCTCTCGTTAATCCATTCA
CE-28S-rRNA_torrent_code4r	CCTCTCTATGGGCAGTCGGTGATAGCACTGTAGTCTCGTTAATCCATTCA
CE-28S-rRNA_torrent_code5r	CCTCTCTATGGGCAGTCGGTGATATCAGACACGTCTCGTTAATCCATTCA
CE-28S-rRNA_torrent_code6r	CCTCTCTATGGGCAGTCGGTGATATATCGCGAGTCTCGTTAATCCATTCA
CE-28S-rRNA_torrent_code7r	CCTCTCTATGGGCAGTCGGTGATCGTGTCTCTATCTCGTTAATCCATTCA

Supplementary Table 3 | Primers used in this study
